# Development and validation of an online emotional intelligence training program

**DOI:** 10.3389/fpsyg.2023.1221817

**Published:** 2023-08-17

**Authors:** Michelle R. Persich Durham, Ryan Smith, Sara Cloonan, Lindsey L. Hildebrand, Rebecca Woods-Lubert, Jeff Skalamera, Sarah M. Berryhill, Karen L. Weihs, Richard D. Lane, John J. B. Allen, Natalie S. Dailey, Anna Alkozei, John R. Vanuk, William D. S. Killgore

**Affiliations:** ^1^Department of Psychiatry, University of Arizona, Tucson, AZ, United States; ^2^Laureate Institute for Brain Research, Tulsa, OK, United States; ^3^Department of Psychology, University of Illinois Chicago, Chicago, IL, United States; ^4^Department of Psychology, University of Arizona, Tucson, AZ, United States

**Keywords:** emotional intelligence, emotional skills, emotional resilience, training program, online resource, MSCEIT, SREIS, TEIQue

## Abstract

**Introduction:**

Emotional intelligence (EI) is associated with a range of positive health, wellbeing, and behavioral outcomes. The present article describes the development and validation of an online training program for increasing EI abilities in adults. The training program was based on theoretical models of emotional functioning and empirical literature on successful approaches for training socioemotional skills and resilience.

**Methods:**

After an initial design, programming, and refinement process, the completed online program was tested for efficacy in a sample of 326 participants (72% female) from the general population. Participants were randomly assigned to complete either the EI training program (*n* = 168) or a matched placebo control training program (*n* = 158). Each program involved 10-12 hours of engaging online content and was completed during either a 1-week (*n* = 175) or 3-week (*n* = 151) period.

**Results:**

Participants who completed the EI training program showed increased scores from pre- to post-training on standard self-report (i.e., trait) measures of EI (relative to placebo), indicating self-perceived improvements in recognizing emotions, understanding emotions, and managing the emotions of others. Moreover, those in the EI training also showed increased scores in standard performance-based (i.e., ability) EI measures, demonstrating an increased ability to strategically use and manage emotions relative to placebo. Improvements to performance measures also remained significantly higher than baseline when measured six months after completing the training. The training was also well-received and described as helpful and engaging.

**Discussion:**

Following a rigorous iterative development process, we created a comprehensive and empirically based online training program that is well-received and engaging. The program reliably improves both trait and ability EI outcomes and gains are sustained up to six months post-training. This program could provide an easy and scalable method for building emotional intelligence in a variety of settings.

## Introduction

Emotions figure prominently in everyday life, yet people differ in their ability to perceive, process, regulate, and utilize emotions in an adaptive manner (Mayer et al., 2000). This individual difference in emotional abilities is captured by the construct of emotional intelligence (EI). There is strong evidence to suggest that EI plays a critical role in desirable social–emotional skills (e.g., leadership, resilience), and is beneficial in several major life domains (Grewal and Salovey, 2006). Due to the positive outcomes associated with EI and the popularity of the construct, much attention has been given to the question of whether EI can be increased through training (Schutte et al., 2013). This question has led to the creation of many EI interventions, particularly in educational and organizational settings. Overall, the evidence from such interventions suggests that EI can be trained, with several meta-analyses reporting moderate increases in EI following intervention (Kotsou et al., 2019; Mattingly and Kraiger, 2019) that also tend to persist across time (Hozdic et al., 2018).

Despite the relative success of previous intervention work, there still remains a need for strong, empirically based EI interventions, and there are remaining questions about the content, design, and administration of such inventions that need to be addressed (Schutte et al., 2013). For instance, differences in theoretical approaches to EI and the particular skills targeted in interventions may impact training outcomes (Mattingly and Kraiger, 2019). In addition, there are various components of EI interventions that require further research, such as online vs. in-person training. Finally, there have been concerns regarding the research design of previous EI intervention studies, including the pervasiveness of small sample sizes and the lack of randomized control designs, which make intervention effects difficult to interpret (Kotsou et al., 2019). Here, we describe the development and validation of an empirically based EI intervention that follows recommended practices (Hozdic et al., 2018) and addresses understudied areas in the EI intervention literature.

### Theoretical approaches to emotional intelligence

A major source of debate and confusion in the EI literature has revolved around how to best conceptualize and measure EI (Roberts et al., 2010). The fundamental contrast is between models that conceptualize EI as an ability and models that conceptualize EI as a trait. The ability-based model defines EI in terms of specific cognitive-emotional skills and measures EI using performance-based metrics that are compared to normative responses (Mayer et al., 2001). The most widely utilized ability-based approach is Mayer & Salovey’s four-branch model of EI (Mayer and Salovey, 1997), which views EI as the ability to (a) accurately perceive emotion, (b) use emotion to facilitate thought, (c) understand emotion, and (d) manage one’s own and others’ emotions. In contrast, the trait-based model defines EI more broadly as a collection of dispositional tendencies and self-perceived abilities (Petrides and Furnham, 2001). The trait-based approach typically utilizes self-report assessments and often includes personality-like characteristics (e.g., empathy, self-esteem, self-motivation) that may fall outside of traditional definitions of performance ability or intelligence (Roberts et al., 2010).

Despite the theoretical concerns and differences between the approaches, meta-analyses show that both trait EI and ability EI tend to increase following focused training interventions (Hozdic et al., 2018; Kotsou et al., 2019; Mattingly and Kraiger, 2019). However, many intervention studies only include either ability-based EI *or* trait EI measures as outcomes, making it difficult to make systematic comparisons between the different conceptualizations of EI. This is important because some have noted that the two approaches may be distinct and complementary (Petrides et al., 2007) and that the interpretation of an intervention’s success may depend on the measures used (Mattingly and Kraiger, 2019). Accordingly, it would be useful to systematically assess changes to both ability-and trait-based EI following an EI training intervention (Schutte et al., 2013). Therefore, the present study was designed to assess the effectiveness of the novel EI training program on both theoretical models.

### Intervention content

When creating an EI intervention, careful consideration should be given as to what emotional skills should be targeted and what content should be included in the training. There is a large EI literature from which to draw content when developing an intervention. In addition, there are largely independent but conceptually related traditions such as emotion regulation (Peña-Sarrionandia et al., 2015), mindfulness (Brown and Ryan, 2003), social competence (Halberstadt et al., 2001), and clinical intervention (Barlow et al., 2011) that can supplement and reinforce EI abilities.

Previous training programs have differed widely in the EI content included, and the information chosen likely depends on the target population, purpose, and theoretical approach of the intervention. These previous interventions have taken different approaches to designing intervention content, including comprehensively training a collection of emotional skills (Nelis et al., 2009), focusing solely on single emotional abilities (Herpertz et al., 2016), and targeting areas tangentially related to EI (e.g., leadership; Kruml and Yockey, 2011). Evidence from social–emotional skill interventions suggests that multimodal trainings tend to produce better short-term and long-term outcomes compared to interventions that focus on training a single skill (Beelman et al., 1994). In addition, training programs that are specifically developed to comprehensively target EI appear to be more effective at increasing overall EI than those that only include elements of EI (Kotsou et al., 2019).

### Administration of EI interventions

The application of computer technology and online training approaches continues to evolve rapidly and online training is becoming widely accepted and utilized in many settings. To date, the effectiveness of online EI interventions is fairly understudied, although one investigation found that a hybrid EI intervention was just as effective as traditional face-to-face administration (Kruml and Yockey, 2011). Our own preliminary work suggested that it was possible to improve EI ability using an online program to train specific emotional skills (Alkozei et al., 2019). The ability to provide online alternatives to in-person training should make such interventions more widely accessible and feasible to conduct and would be particularly useful for large-scale training, such as for the military. In addition, an online training program may provide opportunities to include components that increase learning and engagement, such as customized tailoring based on an individual’s responses, interactive scenarios and activities, immediate and personalized feedback to help guide an individual toward achieving program goals, and personal summaries of progress to assist with self-awareness and self-reflection.

A second understudied area in the EI intervention literature is the role of the timing of the training content. Research on learning and retention shows that distributing practice over a longer period tends to be more effective for the long-term retention of information than compressing practice into a shorter timeframe (Cepeda et al., 2006). However, only one study has compared different training schedules of an EI intervention. Kruml and Yockey (2011) compared a 7- and a 16-week leadership class and found no differences in the effect of the scheduling of content distribution on EI skills. If there are no significant benefits related to different training distribution, interventions may be more appropriately designed according to timeframes that maximize recruitment and retention (Coday et al., 2005).

### Research design and practices in EI interventions

Finally, it is worth noting some of the limitations of previously developed interventions. Although a considerable number of EI interventions exist, many have critical flaws (Kotsou et al., 2019). EI interventions often use small sample sizes that are likely underpowered to detect the effect sizes typically reported in meta-analyses, and may artificially inflate the effects (Sterne et al., 2000). Many interventions also do not include an active control group for comparison, raising potential concerns about placebo, group, and/or test–retest effects driving improvements to EI (Kotsou et al., 2019). There is, therefore, a need for adequately powered EI interventions in which participants are randomly assigned to the intervention or an active-control condition.

### Current study objectives and hypotheses

In light of the conceptual questions still to be answered and the limitations of previous EI intervention work, the objective of the present study was to develop, refine, and validate a comprehensive online training program to enhance EI. Critically, the lesson content was thoroughly grounded in empirically-based research on EI and related emotional skills (e.g., emotion regulation), and the training modules were developed and refined through an extensive multiple-iteration process. Once finalized, the intervention was tested against a closely matched placebo control program using a randomized control group design in a large sample of healthy adults. Our primary hypothesis was that individuals assigned to the EI training program would show improvements to both ability-and trait-based EI scores relative to those completing a similarly engaging active control training program. As a secondary hypothesis, we tested whether administering the program in a distributed manner (over 3 weeks) would increase the learning and retention of the material relative to a compressed administration of the same content (over 1 week).

## Methods

In the subsequent sections, we describe the development and refinement process and the methods for program validation.

### Phase I: program development and refinement

*Conceptual Development*. The goal of this phase of the project was to develop a training intervention that was grounded in prior research on EI, emotion theory, and skill development. To that end, a panel of experts on emotion theory and clinical psychology (WK, KW, RL, and JA) was assembled to provide input regarding potential ways to improve upon an early pilot version of the program that was described elsewhere (Alkozei et al., 2019). This re-conceptualization and refinement process resulted in a comprehensive program design that covered seven major training domains, including (1) Foundational Knowledge of Emotions, (2) Knowing One’s Own Emotions, (3) Motivation, (4) Managing Emotions, (5) Knowing others’ Emotions, (6) Managing Others’ Emotions, and (7) Empathy. Detailed descriptions of the EIT program goals and objectives are provided in Table 1. As shown in the Supplementary material, the refined program was extensively based on the MSCEIT model, but this revised and significantly expanded version also incorporated additional concepts from contemporary emotion theory and research, such as emotion regulation, mindfulness, social intelligence, and countering cognitive distortions. It also provided an overarching evidence-based framework for understanding the causes and effects of emotions, with accompanying graphics and interactive activities.

**Table 1:** Content summary for the emotional intelligence training (EIT) program.

Overarching program goal	Objective(s)
Foundational knowledge of emotions	Describe the function and value of experiencing emotions Explain the physiological underpinnings of emotion Discuss how context informs the emotional response
Knowing one’s own emotions	Differentiate emotions within the emotional dictionary Recognize and label their own emotions
Motivation	Remember the potential benefits (interpersonal) of the ability to regulate emotions Remember the potential difficulties due to the inability to regulate emotions
Managing emotions	Demonstrate motivation for practice in mindfulness Recall the components of mindfulness Identify one’s own cognitive distortions Reframe cognitive distortions Recognize the state of mind from which an individual is acting Identify different types of emotion avoidance strategies they use Understand the consequences of emotion avoidance Identify Emotion Driven Behaviors (EDBs) in their life Plan counter behaviors to EDBs
Knowing others’ emotions	Interpret others’ emotions with awareness toward own biases
Managing others’ emotions	Identify the most adaptive and effective reaction for changing another person’s emotions Describe the value of positively changing another person’s emotions
Empathy	Demonstrate empathetic responses Demonstrate motivation to practice empathy

*Content Development and Programming*. The training intervention was designed to be self-paced and involved a wide variety of interactive game-like activities and simulations to develop a broad range of emotional and social abilities that addressed the seven conceptual domains described above. Working closely with a professional educational software development company, we designed and programmed 13 training modules comprising 4 introductory lessons, 5 “core skills” training modules, and 4 extended practice and integration modules. The Supplementary material provides detailed descriptions and example screenshots of each module. The web-based interface allowed for the inclusion of tailored feedback, interactive scenarios, game-like activities, writing prompts, and other elements to foster active involvement and improved retention of material (Ryan and Lauver, 2002; Chi and Wylie, 2014). All materials in the programs were screened by a doctoral-level certified clinical speech-language pathologist (NSD) to ensure that all information was presented at no higher than an eighth-grade reading level. The final program interface was organized into three progressive tiers of training comprising approximately 10–12 h of training content and activities to be completed at a self-directed pace over several days or weeks. The first tier focused on introducing the concept of emotional intelligence and the basics of emotional processes, as informed by leading theories of emotion such as constructivist (Barrett, 2017), functionalist (Lench et al., 2015), and appraisal theories (Moors et al., 2013). The second tier focused on teaching specific skills related to emotional intelligence, as also informed by evidence-based psychotherapeutic approaches for training emotional awareness and emotion regulation skills (Barlow et al., 2011). Finally, the third tier focused on providing opportunities for practice and self-exploration. Detailed descriptions of the individual modules are presented in the Supplementary material.

In conjunction with the development of the primary EIT program, we also developed a matched placebo condition to ensure that control participants received a training program with equal duration, engagement, and difficulty. The control condition, known as the placebo awareness training (PAT) program, included multiple lessons with no emotional content (e.g., introductory-level lessons on science and the environment) but were otherwise comparable to the EIT program. The PAT was similarly organized into a three-tier structure: Introduction to the basics of the scientific process (tier 1), learning about scientific practices and how they can be applied to better understand the external world (tier 2), and opportunities to apply knowledge (tier 3).

*Iterative Refinement*. After the modules were fully designed, completed, and implemented, we tested and refined the programs in an iterative manner with small groups of healthy participants. Participants were enrolled in either the EIT or PAT programs and were instructed to record experiences with glitches and errors, issues with comprehension, and thoughts about the appearance and design of the program. Once a sample of *n* = 40 was achieved, data collection was paused in order to examine and correct any issues that were identified. This process was repeated four times for a total sample of *n* = 159, *M_age_* = 22.69, 64% women. During program testing, we were also able to ask the participants to rate their perceptions of each module. Using a 7-point Likert scale, participants were asked to rate how helpful (1 = extremely unhelpful; 7 = extremely helpful), how engaging (1 = extremely unengaging; 7 = extremely engaging), and their motivation to improve their emotional intelligence (1 = very unmotivated; 7 = very motivated) after completing each module.

### Phase II: program validation

To test our hypotheses that EI training would result in greater increases in ability-based and self-reported (trait) EI scores relative to individuals in the PAT program, and whether administering the program in a distributed manner (over 3 weeks) would increase the learning and retention of the material relative to a compressed administration (over 1 week), we conducted a large randomized trial comparing the EIT program versus the PAT program.

*Participants.* Participants were recruited from a large southwestern university in the United States and the surrounding metropolitan area. The study was generically advertised as an “awareness” training study to avoid expectancy effects associated with emotional skills training. To ensure that our study was adequately powered, we conducted an *a priori* power analysis. Using the effect sizes found for the MSCEIT in the initial pilot study for the EIT program (Alkozei et al., 2019), we found that a sample of *n* = 40 would be sufficient to detect a similar effect size for the interaction of interest (*f* = 0.28). However, for the critical between-group differences between the EIT and PAT training, we would need *n* = 148 per training condition for high power (1−*β* = 0.95) to detect similar effect sizes to the ones found in the pilot study (*f* = 0.21). Given the dynamic and longitudinal nature of the study, we also assumed that we would need to account for levels of attrition in our sample size estimate. Therefore, we aimed to recruit a large sample of *n* = 450 that would easily allow us to achieve this sample size, even after accounting for possible large levels of attrition (e.g., 30%). A total of *n* = 448 participants took part in the baseline assessment session, *M_age_* = 23.72, 72% women, 6.5% Asian, 3.3% Black or African American, 21.0% Latino or Hispanic, 60.7% Caucasian, and 5.8% multiracial. Participants provided written informed consent and were compensated for their participation. The protocol for this study was reviewed and approved by the Institutional Review Board of the University of Arizona and the U.S. Army Human Research Protections Office.

### Measures

The following measures were administered:

*Ability-Based EI*. The MSCEIT (Mayer et al., 2002) was used to assess changes in ability-based EI as a result of the EIT program. The MSCEIT is a 141-item test that measures four branches of EI: perceiving emotions, using emotions to facilitate thought, understanding emotions, and managing emotions (Mayer et al., 2003). The MSCEIT yields raw scores for each of the four branches, as well as an overall EI score. Additionally, the test also provides standardized average scores on each outcome based on a normative mean of 100 and *SD* of 15 derived from a normative sample of age-and sex-matched individuals. MSCEIT scores are quantified based on the match between participants’ answers and the consensus of an independent norming sample. MSCEIT scores were calculated using the general consensus scoring option with adjustments for age and sex (Mayer et al., 2003).

*Self-Reported EI Abilities*. The self-reported emotional intelligence scale (SREIS) was used to assess self-reports of EI abilities. The SREIS is a 19-item scale that was specifically designed to map onto the skills assessed by the MSCEIT (Brackett et al., 2006), which allows for systematic comparisons between ability-based and self-reported EI. The SREIS is rated on a 5-point scale (1 = very inaccurate; 5 = very accurate) and includes an overall EI score as well as five subscales: perceiving emotions, using emotions, understanding emotions, managing one’s own emotions, and managing the emotions of others.

*Trait Emotional Intelligence*. The trait emotional intelligence questionnaire (TEIQue) is a 153-item scale that comprehensively assesses domains related to trait EI (Petrides, 2009). The TEIQue captures 15 facets that are grouped into four factors: well-being, self-control, emotionality, and sociability. The TEIQue also provides a global EI score, which includes the previously described factors, plus the additional facets of adaptability and self-motivation. The TEIQue is rated on a 7-point scale ranging from 1 = completely disagree to 7 = completely agree.

*Program Perceptions*. During the initial program development and iterative refinement stage, we asked participants to rate how helpful, engaging, and motivating they perceived each of the training modules to be. This provided useful information about how participants subjectively experienced the program, and we, therefore, assessed these same subjective perceptions in the validation phase of the study as well. During the post-training assessment session, participants rated how helpful (1 = extremely unhelpful; 7 = extremely helpful), how engaging (1 = extremely unengaging; 7 = extremely engaging), and how motivated they were to improve their emotional intelligence (1 = very unmotivated; 7 = very motivated). In this case, however, participants rated the program as a whole, rather than each individual module.

*Procedure.* Participants first reported to a laboratory where they completed demographics and baseline EI assessments and were randomly assigned to either the PAT or EIT program, and either the compressed (1 week) or the distributed (3 week) training schedule. After completing the program according to the assigned schedule, participants reported back to the laboratory to complete post-training EI assessments. All EI assessments were administered at both time points (pre- and post-training). We also conducted a six-month follow-up assessment to investigate whether the skills developed in the EIT program would demonstrate long-term persistence. It should be noted, however, that the scheduled follow-up assessments happened to align with the onset of the COVID-19 pandemic in March 2020. As the result of a university shutdown and moratorium on in-person research, several modifications were made to the study protocol, including administering all assessments online, adding flexibility in the expected timing for completing the follow-up, and collecting a smaller sample size than desired. A total of *n* = 91 participants completed the 6-month follow-up.

## Results

### Phase I: program development and refinement

**Figure 1:**
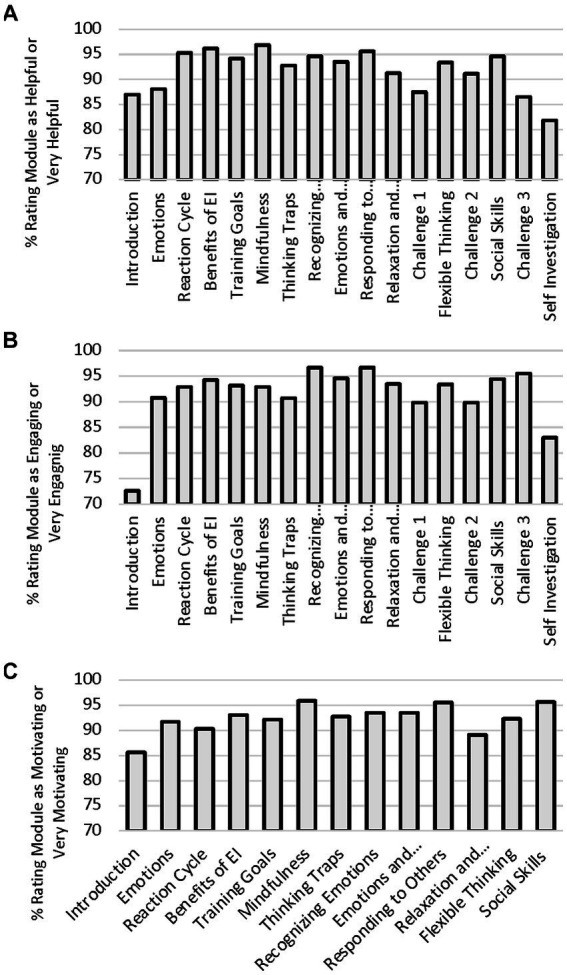
Percentage of participants in the iterative refinement portion of the study rating each modules as helpful or very helpful **(A)**, engaging or very engaging **(B)**, and motivating or very motivating **(C)**.

Through the iterative data collection, we were able to identify and address a number of minor issues (e.g., typos), major issues (e.g., glitches that prevented a person from interacting with the program), problems with clarity (e.g., confusing instructions), and negative personal perceptions (e.g., feedback perceived as condescending). We were also able to determine that participants in the EIT program tended to have positive subjective experiences with the training. As shown in Figure 1, each of the modules tended to be viewed quite favorably. Averaged across all modules, 91.8% of participants perceived the lessons to be helpful or extremely helpful, 91.8% considered lessons to be engaging or very engaging, and 92.4% reported that they felt motivated or very motivated to improve their EI as a result of the lessons.

### Phase II: program validation

*Baseline Descriptive Statistics and Comparison*. Of the initial 448 participants, 122 did not have usable data from the post-training assessments due to scheduling issues, withdrawing from the study, or lack of compliance with the study protocol (27% attrition rate). In total, we were able to produce a high-quality dataset of *n* = 326, with *n* = 168 in the EIT condition (93 compressed; 75 distributed), and *n* = 158 in the PAT condition (82 compressed; 76 distributed). Means, standard deviations, and internal reliability of the assessments at baseline, as well as correlations between all measures, are presented in Table 2. Correlations among the EI measures provided further support for the notion that self-report assessments of EI tend to correlate with other self-reported assessments, but are largely unrelated to performance-based EI measures (Joseph and Newman, 2010). These correlations also revealed that there were some relationships between age, gender, and EI, and that these relationships were mixed. We therefore included both age and gender as covariates in the main analyses.

**Table 2:** Descriptive statistics and item correlations at baseline.

	*M*	*SD*	*α*	1.	2.	3.	4.	5.	6.	7.	8.	9.	10.	11.	12.	13.	14.	15.	16.	17.
**Demographics**																				
1. Age	23.72	5.58	–	–																
2. Gender	72% F	–	–	−0.10*	–															
**MSCEIT (raw)**																				
3. Perceiving	0.5851	0.0671	0.78	−0.18*	0.05*	–														
4. Using	0.4934	0.0616	0.63	0.03	0.06*	0.35*	–													
5. Understanding	0.5526	0.0604	0.72	0.14	−0.01	0.13*	0.37*	–												
6. Managing	0.4185	0.0625	0.69	0.08	0.11*	0.15*	0.39*	0.37*	–											
7. Total	0.5125	0.0430	0.84	0.02	0.08	0.62*	0.77*	0.67*	0.69*	–										
**SREIS**																				
8. Perceiving	3.83	0.65	0.73	0.06	0.08	−0.01	0.00	−0.01	0.08	0.03	–									
9. Using	3.26	0.95	0.77	−0.02	0.18*	−0.04	0.04	0.09	0.04	0.04	0.15*	–								
10. Understanding	3.25	0.89	0.84	0.16*	−0.01	−0.12*	−0.01	0.12	0.08	0.02	0.27*	0.21*	–							
11. Managing (self)	3.78	0.69	0.67	−0.01	−0.19*	0.01	0.04	0.04	0.15*	0.08	0.20*	0.06	0.18*	–						
12. Managing (other)	3.73	0.74	0.78	0.01	0.11*	−0.04	0.01	0.02	0.15*	0.05	0.48*	0.18*	0.30*	0.31*	–					
13. Total	3.59	0.45	0.80	0.07	0.04	−0.07	0.02	0.03	0.16*	0.07	0.67*	0.38*	0.69*	0.57*	0.75*	–				
**TEIQue**																				
14. Well-being	5.53	0.93	0.89	0.00	−0.01	−0.00	0.01	−0.07	0.18*	0.06	0.21*	0.14*	0.09	0.55*	0.35*	0.42*	–			
15. Self-control	4.77	0.80	0.76	0.08	−0.16*	0.07	0.08	−0.08	0.17*	0.11*	0.22*	−0.03	0.08	0.71*	0.30*	0.43*	0.62*	–		
16. Emotionality	5.24	0.77	0.76	0.11*	0.11*	0.01	0.12*	−0.01	0.21*	0.16*	0.40*	0.26*	0.39*	0.35*	0.52*	0.62*	0.50*	0.42*	–	
17. Sociability	4.78	0.78	0.75	0.02	−0.19*	−0.07	−0.03	−0.05	0.06	−0.01	0.39*	0.09*	0.33*	0.43*	0.52*	0.59*	0.46*	0.34*	0.49*	–
18. Total	5.05	0.63	0.90	0.07	−0.05	0.01	0.06	−0.05	0.21*	0.11*	0.38*	0.15*	0.27*	0.66*	0.53*	0.65*	0.84*	0.77*	0.78*	0.70*

Gender is coded 0 = male; 1 = female; correlations with gender are spearman correlations. *p <.05.

Independent sample t-tests showed that participants in the EIT and PAT programs did not differ in terms of age, sex, or EI scores at baseline (see Table 3). We conducted comparisons between the 122 participants who were excluded from the dataset and the 326 participants with complete datasets. Excluded participants did not differ in terms of whether they had been assigned to the EIT or PAT program, *χ*^2^ = 0.23, *p* = 0.628, nor did they differ in terms of demographic variables, *p*s > 0.602. Excluded participants did tend to score lower on the MSCEIT, *t*(440) = −3.58, *p* < 0.001, and TEIQue, *t*(445) = −2.98, *p* = 0.003, and were more likely to have been assigned to the 3-week training schedule, *χ*^2^ = 14.33, *p* < 0.001.

**Table 3:** Differences between groups at baseline.

	EIT		PAT		
	*M*	*SD*	*M*	*SD*	Significance test
**Covariates**					
Age	23.65	5.48	23.79	5.71	*t*(444) = 0.26, *p* = 0.797
Gender (% Female)	71.00%	–	72.86%	–	*Χ^2^* = 0.19, *p* = 0.664
**Baseline outcomes**					
MSCEIT total	0.51	0.05	0.51	0.04	*t*(440) = 0.25, *p* = 0.801
SREIS total	3.59	0.44	3.58	0.47	*t*(442) = 0.10, *p* = 0.923
TEIQue total	5.04	0.64	5.06	0.62	*t*(445) = −0.35, *p* = 0.723

*Program Effects.* Table 4 shows the means and standard deviations of EI scores at baseline and post-training for both the EIT and PAT groups. To evaluate changes in EI, we used linear mixed effects models that could account for both between-group and within-person changes to EI scores. We conducted a series of linear mixed models in R (version 4.2.1), using the “lme4” package, that accounted for differences in age, sex, time, and the interaction between program condition and time. Table 5 shows the fixed effects of the key program condition and time interactions.

**Table 4:** Means and standard deviations of EI variable by time and program condition.

	EIT		PAT	
	Baseline	Post-Tx	Baseline	Post-Tx
**MSCEIT**				
Perceiving	110.64 (16.61)	113.34 (14.94)	110.61 (16.18)	113.27 (14.94)
Using	109.01 (14.58)	111.33 (14.01)	106.66 (14.33)	107.32 (15.26)
Understanding	111.84 (17.99)	115.38 (20.08)	114.23 (20.21)	114.96 (20.97)
Managing	98.32 (12.97)	102.37 (13.43)	98.33 (12.97)	100.28 (13.07)
Total	107.80 (14.27)	112.96 (15.03)	107.41 (13.01)	109.50 (14.29)
**SREIS**				
Perceiving	3.80 (0.67)	3.89 (0.57)	3.86 (0.66)	3.75 (0.69)
Using	3.23 (0.43)	3.30 (0.43)	3.33 (0.46)	3.28 (0.43)
Understanding	3.21 (0.85)	3.60 (0.83)	3.28 (0.93)	3.32 (0.98)
Managing (self)	3.81 (0.63)	3.87 (0.66)	3.85 (0.67)	3.81 (0.73)
Managing (others)	3.73 (0.75)	3.92 (0.66)	3.68 (0.79)	3.73 (0.83)
Total	3.57 (0.43)	3.74 (0.43)	3.61 (0.47)	3.59 (0.51)
**TEIQue**				
Well-being	5.53 (0.88)	5.54 (0.87)	5.64 (0.90)	5.62 (0.93)
Self-control	4.79 (0.77)	4.79 (0.78)	4.87 (0.80)	4.84 (0.84)
Emotionality	5.27 (0.76)	5.35 (0.75)	5.32 (0.76)	5.29 (0.79)
Sociability	4.76 (0.79)	4.82 (0.79)	4.86 (0.78)	4.83 (0.83)
Total	5.06 (0.62)	5.10 (0.61)	5.15 (0.59)	5.12 (0.66)

**Table 5:** Changes in EI scores from baseline to post-training as a function of program condition.

	B [95% CI]	*t*	*p*	*f*2
**MSCEIT**				
Perceiving	0.38 [−2.70, 3.47]	0.24	0.807	0.00
Using	1.78 [−0.76, 4.33]	1.37	0.170	0.02
Understanding	2.31 [−1.03, 5.65]	1.36	0.175	0.02
Managing	2.07 [−0.02, 4.15]	1.95	0.053	0.03
Total	2.90 [0.63, 5.16]	2.52	0.012	0.04
**SREIS**				
Perceiving	0.18 [0.07, 0.29]	3.17	0.002	0.02
Using	0.08 [−0.02, 0.19]	1.52	0.129	0.02
Understanding	0.34 [0.19, 0.48]	4.61	<0.001	0.06
Managing (self)	0.08 [−0.04, 0.20]	1.21	0.226	0.02
Managing (others)	0.13 [0.01, 0.25]	2.04	0.043	0.04
Total	0.17 [0.09, 0.24]	4.36	<0.001	0.04
**TEIQue**				
Well-being	0.02 [−0.08, 0.12]	0.45	0.650	0.00
Self-control	0.02 [−0.08, 0.11]	0.32	0.752	0.00
Emotionality	0.11 [0.001, 0.20]	2.29	0.022	0.02
Sociability	0.08 [−0.01, 0.17]	1.78	0.076	0.01
Total	0.07 [−0.01, 0.14]	1.82	0.069	0.01

Effect sizes represent the proportion of variance explained by the full model relative to a model with covariates only (Lorah, 2018). B represents the unstandardized Beta coefficient.

**Figure 2:**
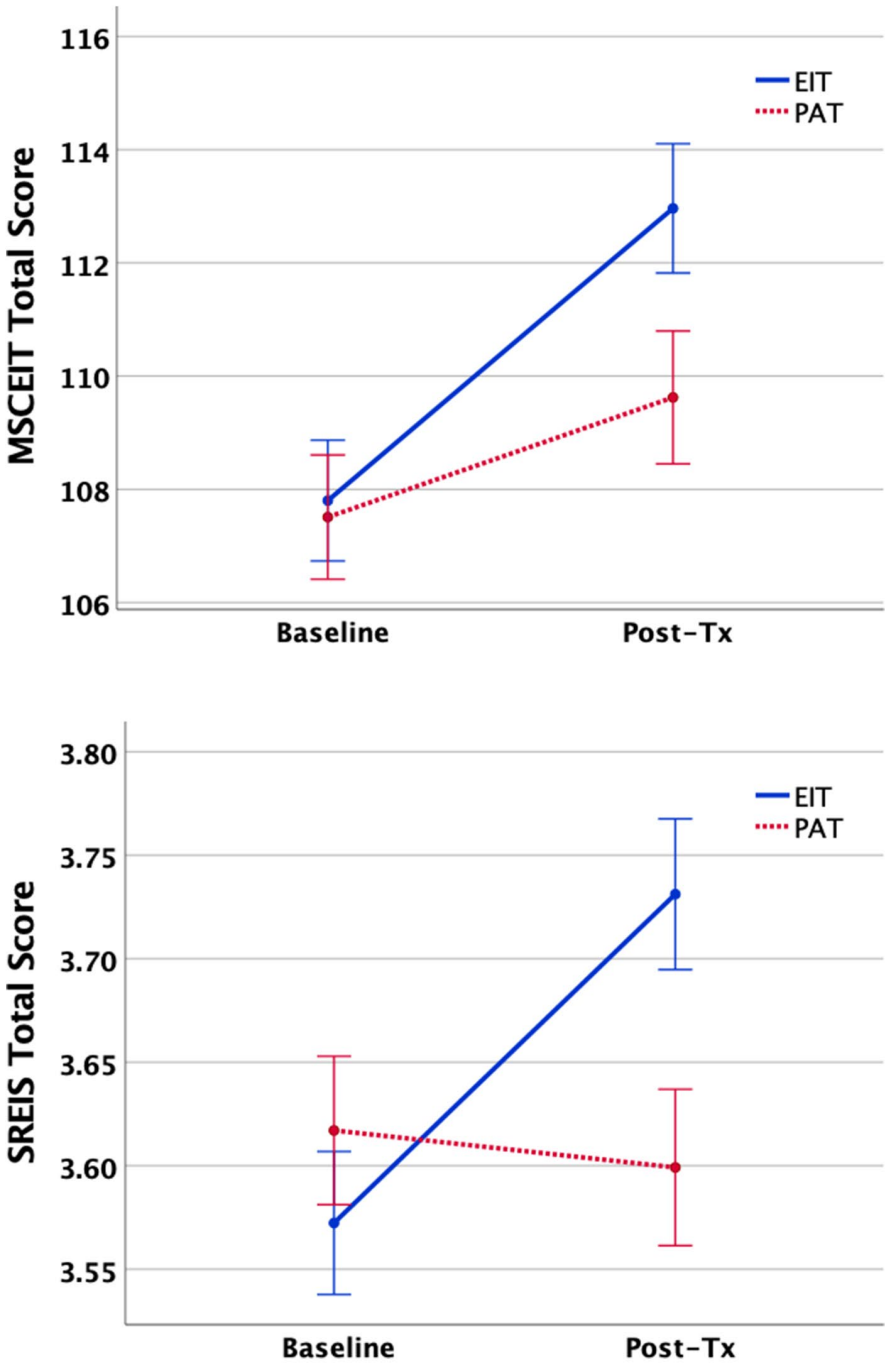
Changes in MSCEIT (top) and SREIS (bottom). Total scores from baseline (T1) to post-training (T2) assessments as a function of training program (mean ± 1SE).

Overall, participants in the EI training program tended to show small improvements to their EI scores relative to participants in the placebo condition [*f*2 = 0.02 is considered small, *f*2 = 0.15 is considered medium, *f*2 = 0.35 is considered large (Cohen, 1992)]. Participants in the EIT program increased their total MSCEIT score by 5.16 points (see Figure 2), *p* < 0.001, moving from what would be considered “competent” (>90 to <110) to “skilled” (>110 to <130) (Mayer et al., 2003), reflecting a medium effect size improvement (*d* = 0.47). Comparatively, the PAT condition improved by 2.11 points (*p* = 0.013), reflecting a small effect size (*d* = 0.21). Although there was no difference at baseline, the EIT group demonstrated significantly higher MSCEIT Total scores at the post-training assessment (*p* = 0.042). Participants also showed improvements in each of the branches of the MSCEIT, but these changes were not statistically significant. Participants in the EIT program showed an increase in their total SREIS scores relative to those in the PAT program (*p* < 0.001), as well as improvements in the subscales related to perceiving (*p* < 0.002), understanding (*p* < 0.001), and managing the emotions of others (*p* = 0.043). Moreover, the increase in SREIS within the EIT group reflected a medium effect size (*d* = 0.46), while the PAT group was associated with a slight and very small decrease in self-reported EI (*d* = −0.05). Finally, the overall TEIQue score also had a marginal trend toward improvement in self-perceived emotional intelligence in the EIT relative to the PAT condition.

For those in the EIT program, we examined relationships among improvements on each of the EI measures (quantified as the within-person difference between post-training and baseline scores for each measure). Changes in the SREIS and TEIQue total scores were correlated, *r* = 0.46, *p* < 0.001, indicating that participants who showed an increase in their SREIS scores also tended to show an increase in the TEIQue scores. However, the level of improvement for MSCEIT was unrelated to changes in SREIS, *r* = 0.07, *p* = 0.349, and the TEIQue, *r* = 0.08, *p* = 0.314. Further examination of the parallel MSCEIT and SREIS subscales also did not find significant correlations between performance-based and self-reported improvements in perceiving, *r* = 0.001, *p* = 0.993, using, *r* = 0.12, *p* = 0.165, understanding, *r* = 0.09, *p* = 0.231, and managing emotion, *r* = 0.12, *p* = 0.130 (SREIS managing self) and *r* = 0.01, *p* = 0.902 (SREIS managing others).

*Effects of Training Distribution Schedule.* One of the study aims was to investigate whether distributing the training across 3 weeks would result in better learning and retention when compared to a compressed 1-week training. Linear mixed effects models with an added condition × time × distribution term revealed that only the SREIS perceiving subscale showed a significant difference, *t* = 2.69*, p* = 0.008, such that the effect of the EIT program was stronger in the distributed (i.e., 3 week) training schedule versus the compressed (i.e., 1 week) schedule only for that outcome variable. No other significant effects were observed, with *p*-values ranging from *p* = 0.073–0.915. We therefore conclude that the distribution of training did not affect the post-training outcomes.

**Figure 3:**
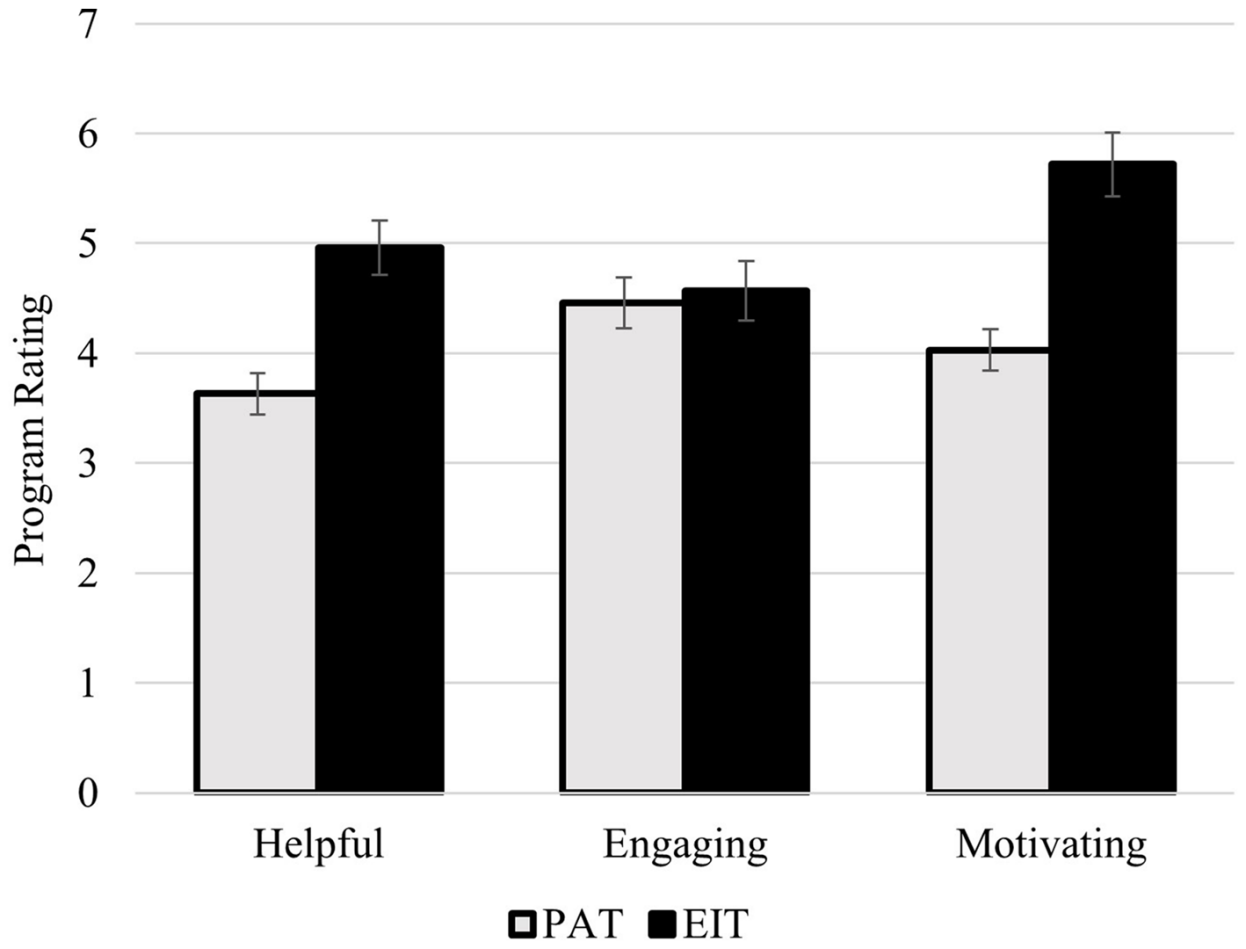
Average program ratings for the placebo awareness training (PAT) and the emotional intelligence training (EIT) programs.

*Program Perceptions*. During the post-training assessment session, we asked participants to report on how helpful, engaging, and motivating the programs were. As shown in Figure 3, the overall EIT program was rated positively, with averages above the midpoint of the 7-point scale for each of the questions. Participants tended to view both the EIT and the placebo programs as similarly engaging, *t*(300) = 0.59, *p* = 0.553. However, participants in the EIT program perceived the training to be more helpful, *t*(300) = 8.33, *p* < 0.001, and were more motivated to improve their emotional intelligence, *t*(300) = 9.69, *p* < 0.001, relative to participants in the PAT program. These ratings were also highly correlated, such that participants who found their program to be helpful also tended to find it engaging (*r* = 0.56, *p* < 0.001) and were motivated to improve (*r* = 0.72, *p* < 0.001); and participants who found their program engaging were also motivated to improve (*r* = 0.41, *p* < 0.001).

*Long-Term Persistence of EI Improvements.* As initially designed, the six-month follow-up was intended to be a key component of the main analyses. Unfortunately, the COVID-19 pandemic emerged contemporaneously with the timing of the 6-month follow-ups, potentially affecting the emotional and mental health functioning of the participants and leading to a disruption in data collection. Therefore, due to COVID-related changes to the study protocol and the general climate in which participants completed the assessments, we cannot be fully confident that the following results represent an accurate evaluation of the long-term effectiveness of the EIT program. We therefore present the 6-month follow-up analyses separately and interpret these findings cautiously.

In total, *n* = 91 participants completed the 6-month follow-up with *n* = 37 in the PAT condition (22 compressed; 15 distributed), and *n* = 54 in the EIT condition (25 compressed, 29 distributed). There were no baseline differences found between the 91 participants who completed the 6-month follow-up assessments and the 357 participants who did not in terms of age, gender, training distribution condition, SREIS, and TEIQue total scores (all *p*s > 0.522). The number of participants in each condition did not differ significantly, *χ*^2^ = 2.86, *p* = 0.099, but participants who completed the 6-month follow-up had higher MSCEIT total scores at baseline (*M* = 109.88 *SD* = 16.21) than the participants who did not complete the 6-month follow-up (*M* = 105.25, *SD* = 13.14), *t* = 2.86, *p* = 0.004.

To analyze the long-term effects of training, we ran linear mixed effects models that accounted for differences in age, sex, time, and the interaction between program condition and time. There was a significant group × time interaction for the MSCEIT total score, *t* = 2.57*, p* = 0.011*, f^2^* = 0.03*. Post hoc* analyses revealed that the MSCEIT scores of participants in the EIT program were significantly higher than they were at baseline, at Time 2, *t* = 5.99, *p* < 0.001, *d* = 0.47, and marginally higher at Time 3, *t* = 1.76, *p* = 0.083, *d* = 0.24, suggesting that the skills trained in the program were modestly retained across an extended period, despite the emergence of the worldwide COVID-19 pandemic. There was also a significant group × time interaction for the SREIS, *t* = 2.40*, p* = 0.017*, f^2^* = 0.01. However, *post hoc* analyses revealed that this effect was driven by increases in SREIS scores for EIT participants at Time 2, and their SREIS total scores were no longer significantly different than baseline at Time 3, *t* = 0.95, *p* = 0.349, *d* = 0.13. Finally, there was a marginally significant group × time interaction for the TEIQue, *t* = 1.95*, p* = 0.052*, f^2^* = 0.01, but these scores on the TEIQue did not significantly differ from baseline at Time 3, *t* = 1.57, *p* = 0.123, *d* = 0.21. There was no effect of training distribution on any of these scores, *p*s > 0.320.

## Discussion

The goal of this study was to develop an empirically based, online training program and investigate its efficacy in building and sustaining emotional intelligence. We created a roughly 10-h web-based training program based on the MSCEIT model of EI and theoretically related traditions such as emotion regulation, mindfulness, social skills, and clinical practice then investigated the efficacy of the program using a large sample and a randomized control trial design. As hypothesized, participants who completed the EI training program improved their total ability and self-reported EI scores relative to those in the placebo condition. Overall, participants in the EIT program demonstrated over a five-point increase in their ability-based EI, to elevate the mean performance from what the MSCEIT manual describes as the “competent” to the “skilled” range (Mayer et al., 2003), consistent with a medium effect size improvement following training. Participants in the EIT program also showed increases in their self-reported EI abilities, including specific improvements in perceiving emotion, understanding emotions, and managing the emotions of others, again reflecting a medium effect size improvement. The EIT program content was rated as engaging and helpful in improving EI skills and participants reported that they were motivated to continue their emotional growth upon completion relative to the PAT group. These data suggest that the presently reported online EIT program is effective at developing a range of emotional intelligence capacities and skills and that these improvements show trend-level retention over time, even during the emergence of a worldwide pandemic crisis. The present study extends the field of research on EI interventions in a number of ways. To our knowledge, this is the first placebo-controlled study to show that a fully online EI training program can reliably increase EI scores in a large study of adults. In addition, the study design considered criticisms of previous EI interventions and incorporated numerous enhancements to counter those prior limitations. The study was adequately powered to detect small to medium effects and represents the only known large-scale, randomized, placebo-controlled, empirically-based intervention study that demonstrates the efficacy and validity of a comprehensive online EI training program (Kotsou et al., 2019).

An important advancement provided by the present study was our inclusion of multiple assessments that measured both ability-based and trait EI to allow for systematic comparisons between the two theoretical approaches to EI. We found that the EI training resulted in overall improvements to both ability-based EI and self-reported EI. Overall, the EIT program was associated with increases in Total EI scores on the MSCEIT, the most well-established and widely used ability metric of EI. The total score represents a composite index of EI abilities that is broadly representative of an individual’s ability to detect emotional information from people and situations, understand how this information relates to contexts and goals, use that information to enhance thought processes, and effectively modulate emotions in oneself and others appropriately. In particular, the EIT program was most effective at improving the ability of participants to manage and control emotional responses based on consideration of the situational context, a key component of social–emotional success (Mayer et al., 2002). The EIT program also enhanced Total EI scores on the SREIS (Brackett et al., 2006), a self-report measure of EI that is designed to measure the same content domains as the MSCEIT. Participants showed significant increases in their self-reported skills in perceiving their own emotions and the emotions of others, their perceived ability to understand the complexities of emotions (e.g., emotional antecedents; how emotions emerge over time), and their self-described ability to modulate the emotions of other people. Finally, the EIT program showed a marginally significant improvement in the TEIQue Total score, which measures trait EI. Overall, the program was effective at significantly improving the trait of Emotionality, which includes an individual’s self-perceptions of their emotional empathy, perception, and expression, and quality of relationships. The TEIQue also showed a marginally reliable improvement among those who completed the EIT on the factor of Sociability, which includes the self-perceived ability to successfully manage emotions, be assertive, and be aware of social cues and situations. Together, these findings suggest that the EIT program was successful at improving EI as defined by several conceptual models.

Interestingly, while the EIT program was effective at improving both ability and self-reported EI, within individuals overall improvements on the MSCEIT and SREIS were uncorrelated. This is consistent with prior work suggesting that trait and ability EI are only weakly correlated with one another, if at all (Webb et al., 2013). Essentially, it appeared as though improvements to a person’s measured EI abilities might not have necessarily translated into self-reported EI, and a person could have self-reported increases in EI without necessarily showing improvements in EI performance in the associated domain. Future research may want to investigate whether improvements to ability-based versus self-reported EI systematically produce differential effects or interact to predict consequential outcomes.

We also found differences in how improvements to the different EI metrics were maintained over time (although the influence of COVID-19 on this follow-up assessment makes it difficult to confidently interpret these findings). We found that increases in the MSCEIT score were marginally higher than the baseline 6 months following the EI training, whereas scores on the two self-reported EI assessments had returned to baseline levels. Overall, emotional traits should be fairly stable, but trait-related behaviors and self-perceptions can momentarily fluctuate based on context (Fleeson, 2001). Participants may have experienced an increase in their perceptions of emotional competence immediately after completing a training program specifically designed to increase emotional skills, but then ultimately returned to their typical self-perceptions once that situational influence had been removed. Conversely, ability-based EI is more strongly related to standard cognitive intelligence (Webb et al., 2013). Accordingly, emotional abilities that were learned during the training program should be more likely to persist, even after the contextual influence of the training had faded. These findings are consistent with other data from this project that showed that the EIT program significantly improved clinical scores of depression, anxiety, and suicidal ideation compared to the PAT program 6 months after training, just as the COVID-19 pandemic and associated nationwide lockdowns had emerged (Persich et al., 2021). Such findings suggest that not only is the program effective at sustaining EI skills, but that these skills were protective of mental health in a time of real-life crisis.

We also tested a second hypothesis related to the distribution schedule for completing the training content. As previous research on learning models raised the possibility that distributing training over longer periods of time could help participants learn and retain the intervention material more effectively than compressing the content into a shorter timeframe (Cepeda et al., 2006), we had hypothesized that an extended period for completing the training would enhance learning and retention of the EI skills. However, we did not find differences in EI improvement between the compressed (1 week) and distributed (3 week) training conditions. These findings are similar to Kruml and Yockey (2011), who also did not find differences between a 7- and a 16-week intervention. An important implication of this outcome is that it appears that the program can be used flexibly over time (i.e., either distributed or compressed) with similar outcomes overall. However, another important outcome relating to the training duration was that participants in the distributed condition demonstrated a significantly greater attrition rate between pre-and post-training assessments that the compressed training. It is possible that the extended timeframe increased the perceived burden on participants or that they lost interest when the training schedule was too long. Given that we did not find a benefit in extending the timeframe, future studies, and clinical applications may want to consider choosing timeframes that maximize recruitment and retention efforts, as well as testing the efficacy of flexible administration strategies.

Overall, the present study clearly shows that the EIT program produced improvements in several relevant EI metrics. However, we did not find significant results for all subscales and branches of the outcome measures, and the effects were smaller than what has typically been reported in published research using in-person training approaches (Hozdic et al., 2018; Mattingly and Kraiger, 2019). This may be a limitation of the study. These small effects could be attributable to the EIT program’s online format, the program content, or the relatively healthy and emotionally intelligent sample, and more research should be done to investigate whether these effects can be strengthened. However, it is also possible that the smaller effects were attributable to the more stringent and highly controlled study design, and the effects of EI interventions may actually be smaller than typically reported. For instance, participants in the EIT program showed improvements across all MSCEIT branches, but the PAT participants often showed increases as well. This may indicate that other factors (e.g., practice effects, attention effects, regression to the mean) could have contributed to observed improvements in EI following intervention efforts. Importantly, many EI intervention studies have not included an active control group to account for these factors, and it is therefore possible that the effects of EI interventions in published literature have been artificially inflated (Kotsou et al., 2019) due to the lack of appropriate control training conditions. We encourage those interested in developing EI interventions to adopt more rigorous tests and controls, as done here, in order to determine the true effect size of these interventions. Nonetheless, small to medium effect sizes could have meaningful effects when training interventions are implemented at a large scale. Even a 5-point increase in EI could have a tangible and meaningful effect across a large population, potentially influencing relationship quality, wellbeing, and mental health in meaningful ways.

The present study was designed as an initial validation of our novel EI intervention to show that teaching EI skills can result in an increase in quantified levels of EI. With evidence to suggest that the program can increase EI, it would be useful to continue investigating the generalizability of the program in relation to specific populations and external outcomes. This initial validation study tested the effectiveness of the EI intervention in a relatively healthy sample who tended to have above-average scores on each of the three EI measures assessed. It would be useful to extend the present research by investigating the intervention’s effectiveness in specialized samples; such as individuals with poor social–emotional functioning, individuals with clinical disorders characterized by emotional difficulties, or individuals in emotionally demanding roles such as the military or first responders (Joseph and Newman, 2010). In addition, although it is important to establish the effect of training on EI scores, the ultimate goal of an intervention is to show that it improves meaningful intrapersonal and interpersonal outcomes. Future research should test the effect of the program on outcomes such as adaptive behavior in emotionally demanding tasks, successful functioning in work, school, and relationships, as well as long-term well-being and mental health.

## Conclusion

We have successfully developed and validated a novel online EI training program based on well-established theoretical models of emotional intelligence that incorporates empirically supported training content and methods. Our study design addressed several methodological limitations of prior studies of EI training programs. This large, randomized placebo-controlled validation study demonstrated that the program is well accepted and positively received by participants, significantly improves EI scores on both trait and ability measures, is robust to variations in the training schedule, and shows a trend toward sustaining EI skills up to 6 months post-training, even during the emotional challenges posed by the start of a worldwide pandemic crisis and nationwide lockdown orders. With further validation, this approach could provide a standardized and scalable training method for building critical emotional intelligence skills.

## Data availability statement

The raw data supporting the conclusions of this article will be made available by the authors, without undue reservation.

## Ethics statement

The studies involving human participants were reviewed and approved by University of Arizona Institutional Review Board. The patients/participants provided their written informed consent to participate in this study.

## Author contributions

MD carried out the primary data organization and statistical analysis, wrote the initial draft of the manuscript, and contributed to revisions. RS contributed equally to the initial drafting of the manuscript and contributed to the initial study design and development of the EIT materials. SC, RW-L, JS, and SB executed data collection, organized the study database, and also reviewed and edited sections of the manuscript. LH reviewed and edited versions of the manuscript. KW, RL, and JA contributed to the initial conceptualization and redesign of the EIT materials and edited and reviewed drafts of the manuscript. ND, AA, and JV contributed to data collection and statistical analysis, and reviewed drafts of the manuscript. WK was responsible for the initial conceptualization and design of the project, obtaining the research funding, provided oversight of the EIT program design, data collection, statistical analysis and interpretation of the findings, and contributed to initial drafting and revisions of the manuscript. All authors contributed to the article and approved the submitted version.

## Funding

The study was supported by funding from the U.S. Army Medical Research and Development Command (W81XWH-16-1-0062) to WK.

## Conflict of interest

The authors declare that the research was conducted in the absence of any commercial or financial relationships that could be construed as a potential conflict of interest.

## Publisher’s note

All claims expressed in this article are solely those of the authors and do not necessarily represent those of their affiliated organizations, or those of the publisher, the editors and the reviewers. Any product that may be evaluated in this article, or claim that may be made by its manufacturer, is not guaranteed or endorsed by the publisher.
