# Supplementary material for: Development and validation of an online emotional intelligence training program
Source: Front Psychol. 2023 Aug 17;14:1221817. doi: 10.3389/fpsyg.2023.1221817 (PMC10470876; doi:10.3389/fpsyg.2023.1221817)
Supplement: Supplementary file 1 [file Data_Sheet_1.docx]

**Module 1 (Part 1): Introduction**


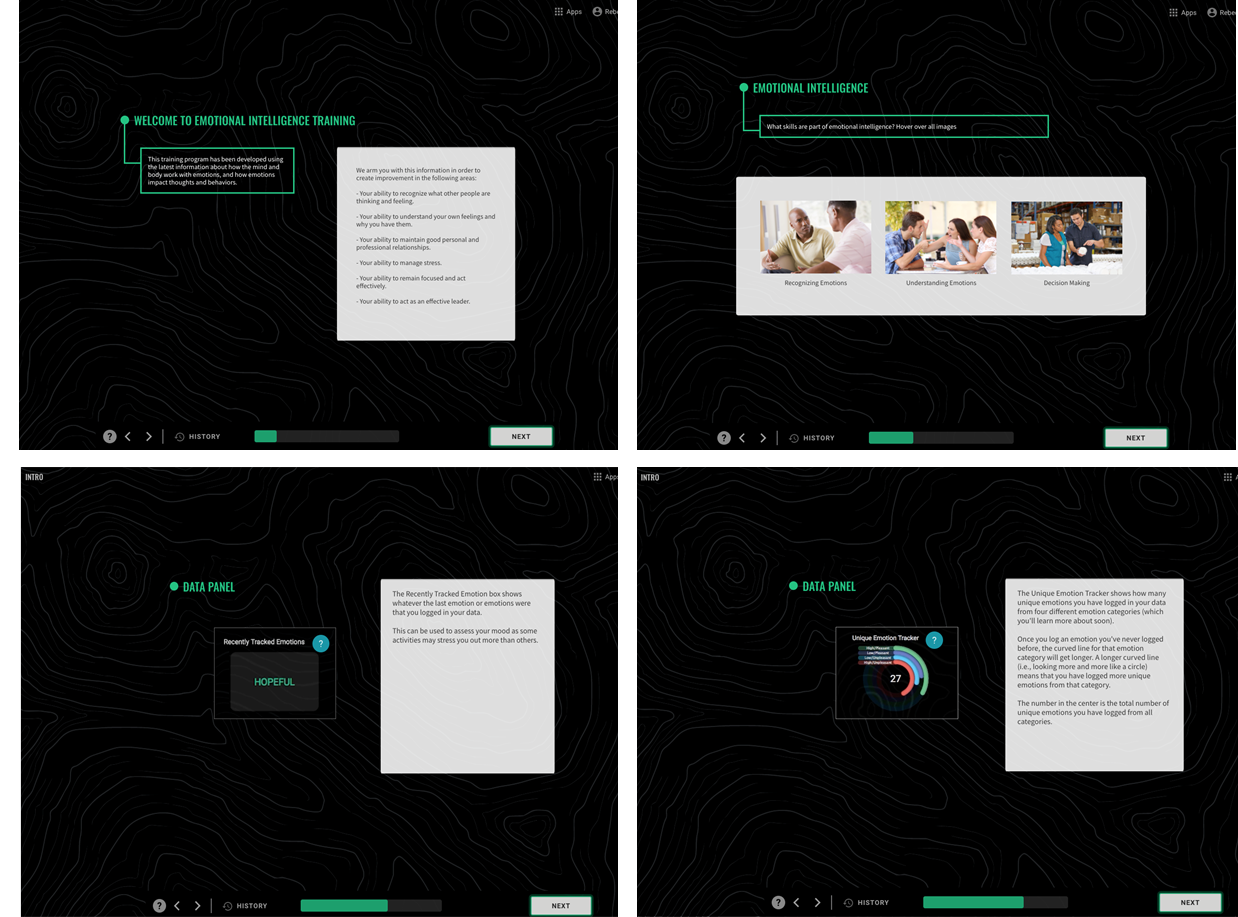


*Figure 1*. Introduction to the program and description of the training goals (top left); Interactive descriptions of the skills that make up EI (top right); Description of tools for tracking emotions that will be used in the program (bottom images).

Images reproduced with permission from Monkey Business Images/shutterstock.com and Antonio Guillem/shutterstock.com.

**Module Aims**: This module introduces the participant to the concept of emotional intelligence and some essentials of the program.

**Targeted Knowledge, Skills, and Abilities**: Developing a basic understanding of EI and the skills related to EI, a basic understanding of how EI can be beneficial, a basic understanding of the general program interface, structure and goals of the program, and a familiarity with the tools that will be used throughout the program.

**Description of Activities**: Welcome, introduction to emotional intelligence, video describing structure and goals of the program, overview of the tools used in the program

**Average Completion Time*:** 8 minutes

**Schedule:** Day 1 (Compressed); Day 1 (Distributed)

* Average completion time based on the initial testing and development group described in Study 1 of current paper

**Module 1 (Part 2): Emotions**


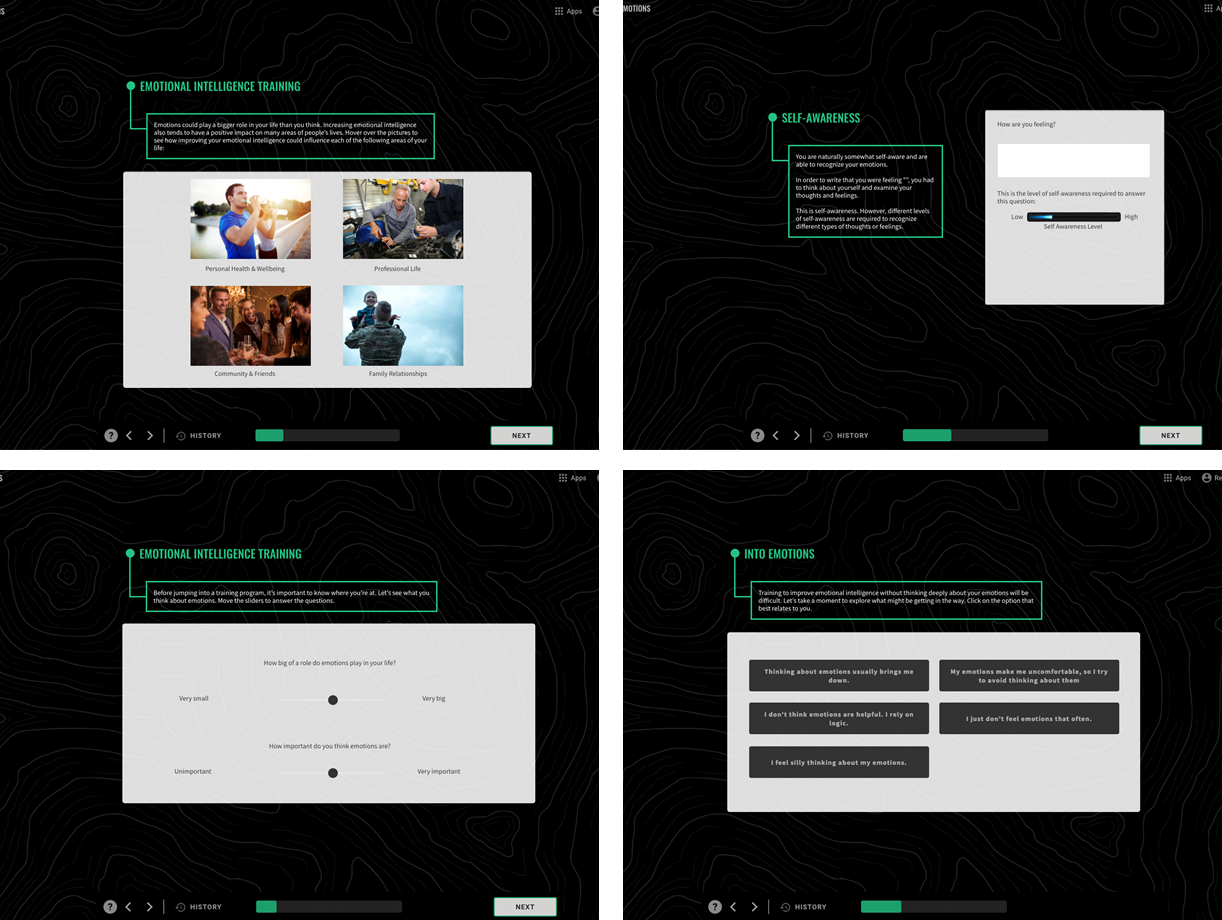


*Figure 2*. Interactive descriptions of how EI can benefit one’s life in various domains (top left); Introduction to the concept of self-awareness (top right); Assessing initial beliefs about emotions (bottom left); Addressing initial beliefs about emotions (bottom right).

Images reproduced with permission from Ground Picture/shutterstock.com; Monkey Business Images/shutterstock.com; goodluz/shutterstock.com and NDAB Creativity/shutterstock.com.

**Module Aims**: This module assesses initial beliefs about emotions and current emotional skills.

**Targeted Knowledge, Skills, and Abilities**: Understanding how EI can be beneficial, dispelling myths and negative impressions of emotions, developing self-awareness skills to recognize and explore thoughts, emotions, body reactions, and behaviors.

**Description of Activities**: Reflection on previous experience with training programs, reflection on current beliefs about emotions, tailored feedback based on initial beliefs, activity to introduce concept of self-awareness, introduction to the connections between situations, interpretations, emotions, and behaviors.

**Average Completion Time:** 9 minutes

**Schedule:** Day 1 (Compressed); Day 1 (Distributed)

**Module 2: The Reaction Cycle**


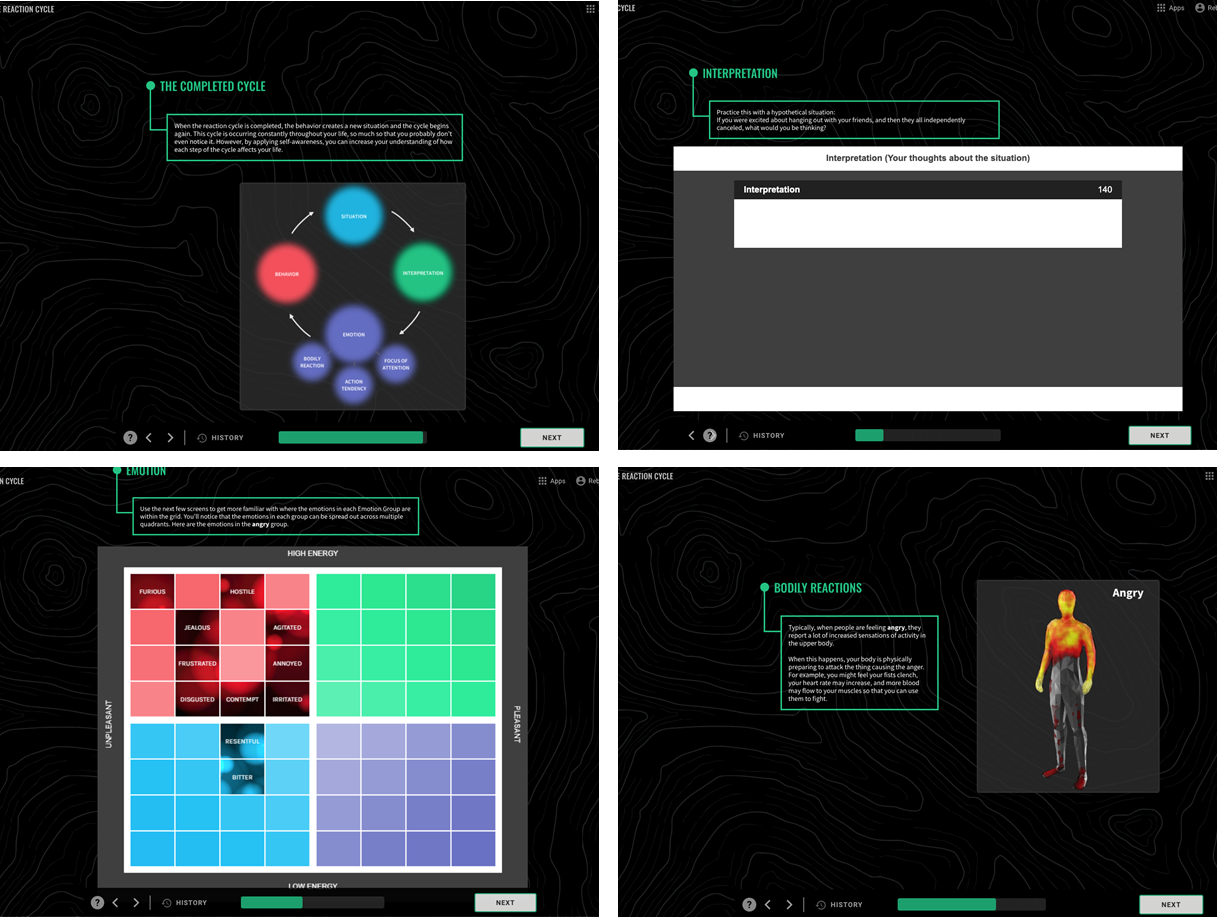


*Figure 3*. Illustration of the reaction cycle (top left); Interpreting a situation (top right); Depiction of different emotions that can be classified as “angry” and how they differ based on arousal and valence (bottom left: Russel, 1980); Depiction of the bodily sensation of anger (bottom right: Nummenmaa, Glerean, Hari, & Hietanen, 2013).

All images and content were produced by the University of Arizona.

**Module Aims**: This module covers why understanding emotion can be helpful in maintaining a stable, healthy state and staying focused to accomplish goals. The main focus is teaching something called the ‘reaction cycle’ and helping the participant notice this in their own life.

**Targeted Knowledge, Skills, and Abilities**: Defining situations, accurately interpreting situations, how to differentiate and categorize emotions, recognizing bodily reactions to emotion, recognizing how emotions can change the focus of attention, connecting the situations, interpretations, and emotions to behavior.

**Description of Activities**: Videos describing each element of the emotion tracker, practice with using the emotion tracker (e.g., making interpretations, differentiating emotions using the emotion grid, filling in a bodily sensation figure).

**Average Completion Time:** 1 hour

**Schedule:** Day 1 (Compressed); Day 2 (Distributed)

**Module 3: The Benefits of Emotional Intelligence**


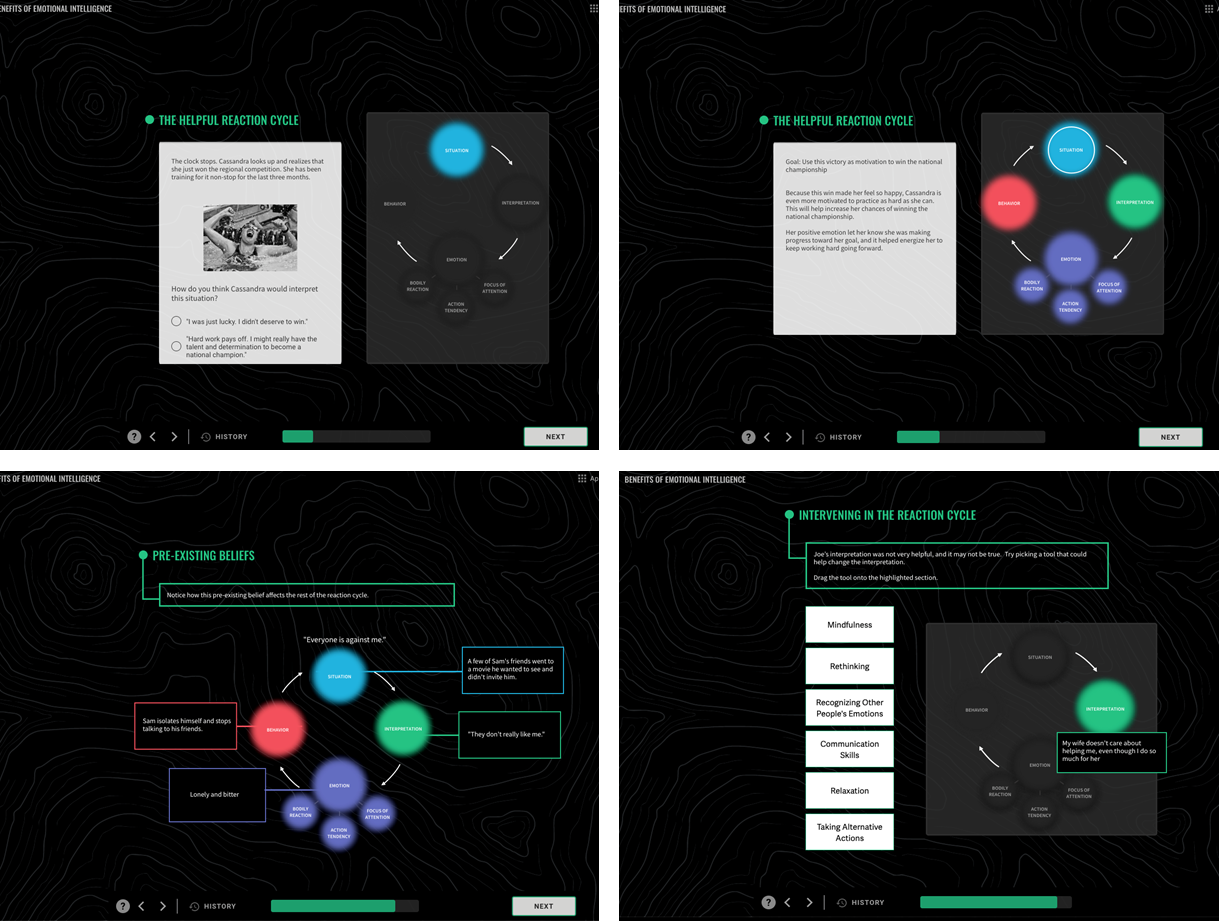


*Figure 4*. Scenario depicting how an emotional reaction can facilitate positive behaviors that meet long-term goals (top images); scenario depicting how pre-existing beliefs can impact each stage of the reaction cycle (bottom left); interactive graphic illustrating how emotional skills can prevent negative loops (bottom right).

Images reproduced with permission from Pixabay.

**Module Aims**: This module describes the benefits of increasing emotional intelligence and how it can help maintain healthy relationships, improve leadership skills, maintain attention on completion of important goals, among others.

**Targeted knowledge, skills, and abilities**: Learning about when emotions can be helpful or harmful, understanding how harmful emotional reactions can contribute to negative reaction cycles, identifying pre-existing beliefs and the role they play in emotional reactions, how emotional skills can prevent negative reaction cycles

**Description of Activities:** Interactive scenarios of emotional reactions in different situations with both positive and negative outcomes, examples of how emotional skills can help intervene in negative cycles.

**Average Completion Time**: 30 minutes

**Schedule:** Day 2 (Compressed); Day 3 (Distributed)

**Module 4: Training Goals**


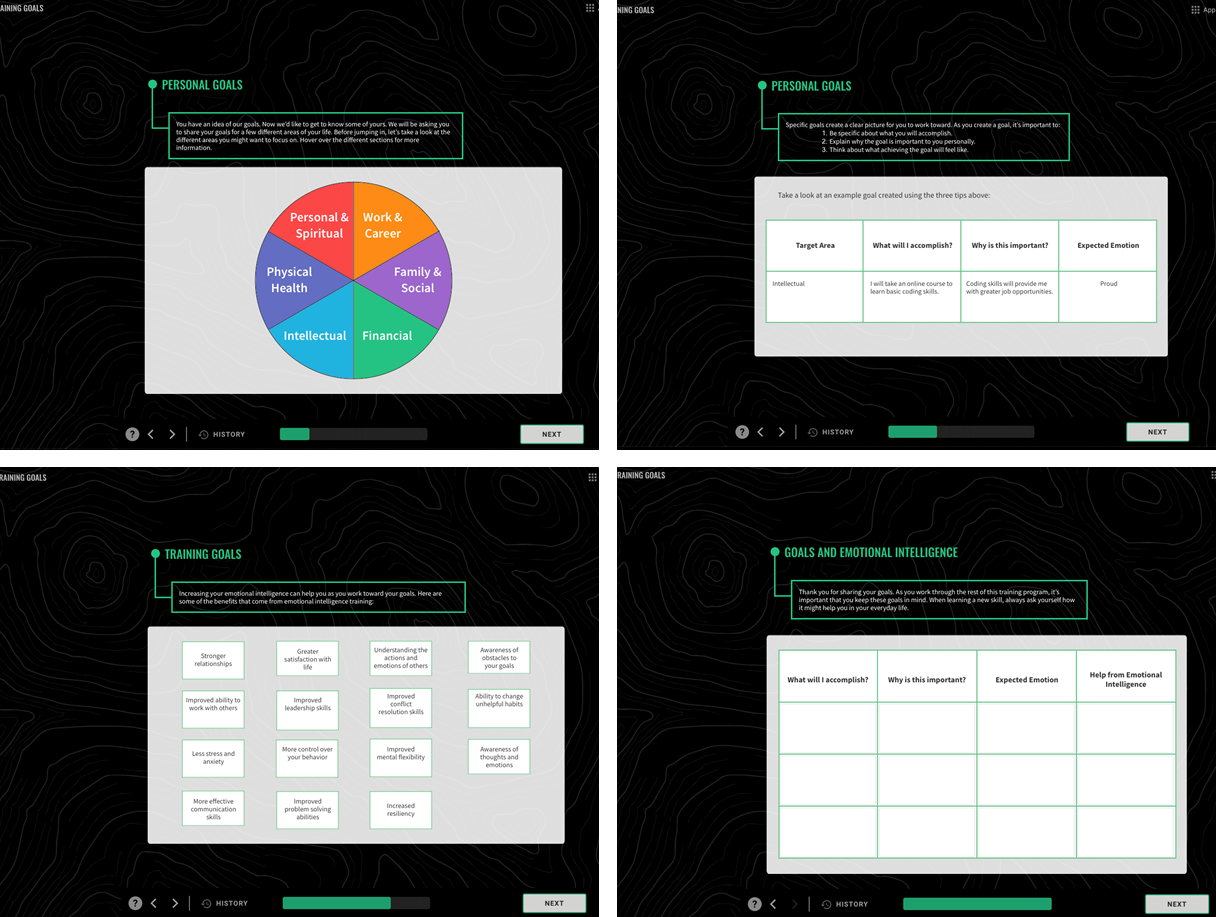


*Figure 5*. Major areas of life for which people often have goals (top left); example of a specific goal (top right); ways in which EI can help with goals (bottom left); interactive goal activity for participants to apply what they learned (bottom right).

All images and content were produced by the University of Arizona.

**Module Aims**: This module asks the participant to identify the skills that matter most to them, to identify the specific benefits they expect from developing them, and to set their own goals.

**Targeted Knowledge, Skills, and Abilities**: Learning how to articulate specific and meaningful goals, understanding how EI can assist with achieving important goals and improvements to key areas of life

**Description of Activities**: Overview of major goals areas and how to create a specific goal, application practice where participants generate three personal goals using the programs guidelines.

**Average Completion Time**: 12 minutes

**Schedule:** Day 2 (Compressed); Day 4 (Distributed)

**Module 5: Mindfulness**


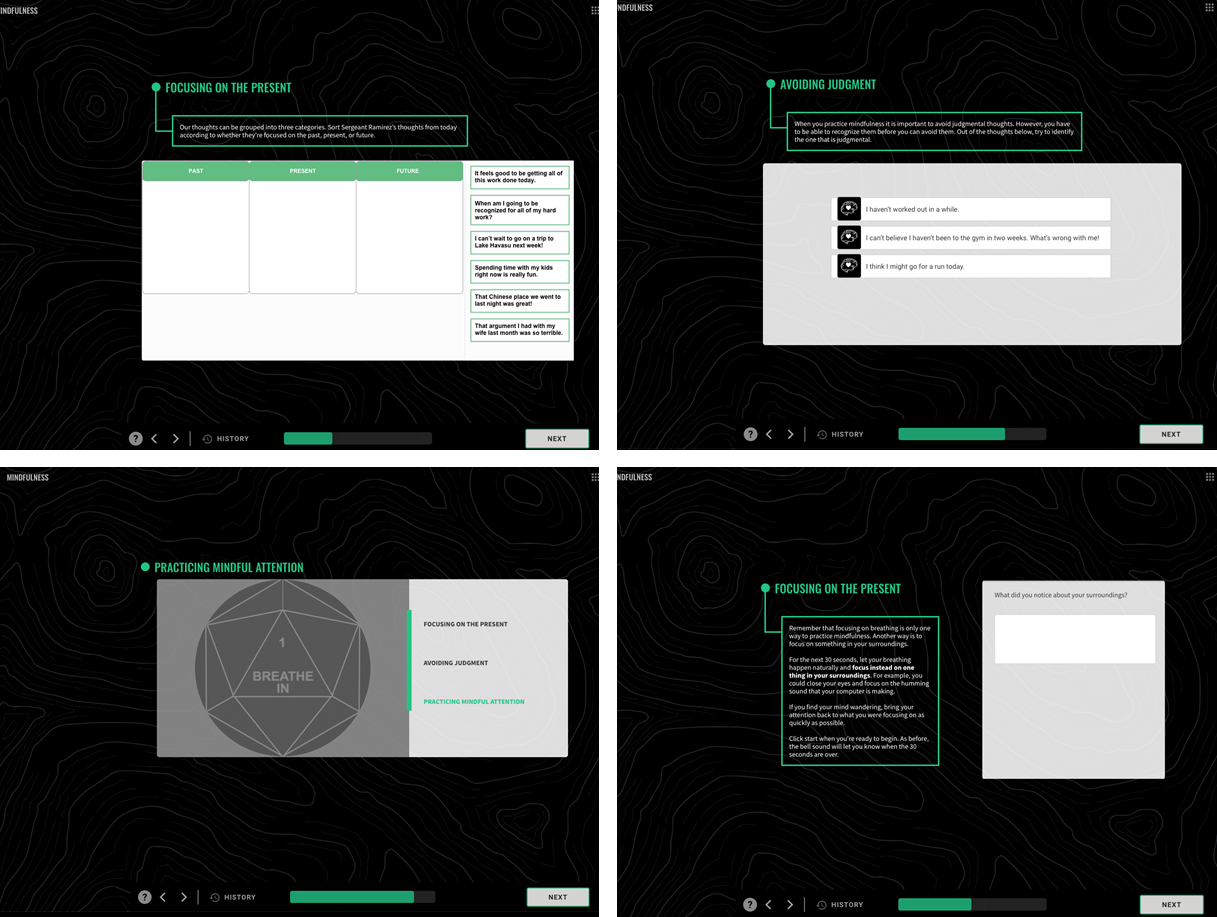


*Figure 6*. Interactive activity classifying thoughts as past, future, or present focused (top left); identifying judgmental thoughts (top right); breathing exercise (bottom left); reflection activity following a breathing exercise (bottom right).

All images and content were produced by the University of Arizona.

**Module Aims**: This module teaches and allows the participant to practice an effective emotion regulation technique called mindfulness.

**Targeted Knowledge, Skills, and Abilities**: Focusing on the present moment, recognizing and avoiding judgmental thoughts, mindful breathing, body awareness

**Description of Activities**: Opening emotion tracker activity, overview of mindfulness and how it fits in the reaction cycle, activity categorizing thoughts as past, present, or future focused, series of 30-second breathing exercises to focus on thoughts, surroundings, and bodily sensations, activity identifying judgmental and non-judgmental thoughts, 5-minute guided mindful attention exercise, self-reflection prompts at the end of each exercise and activity

**Average Completion Time**: 41 min

**Schedule:** Day 3 (Compressed); Day 8 (Distributed)

**Module 6: Thinking Traps**


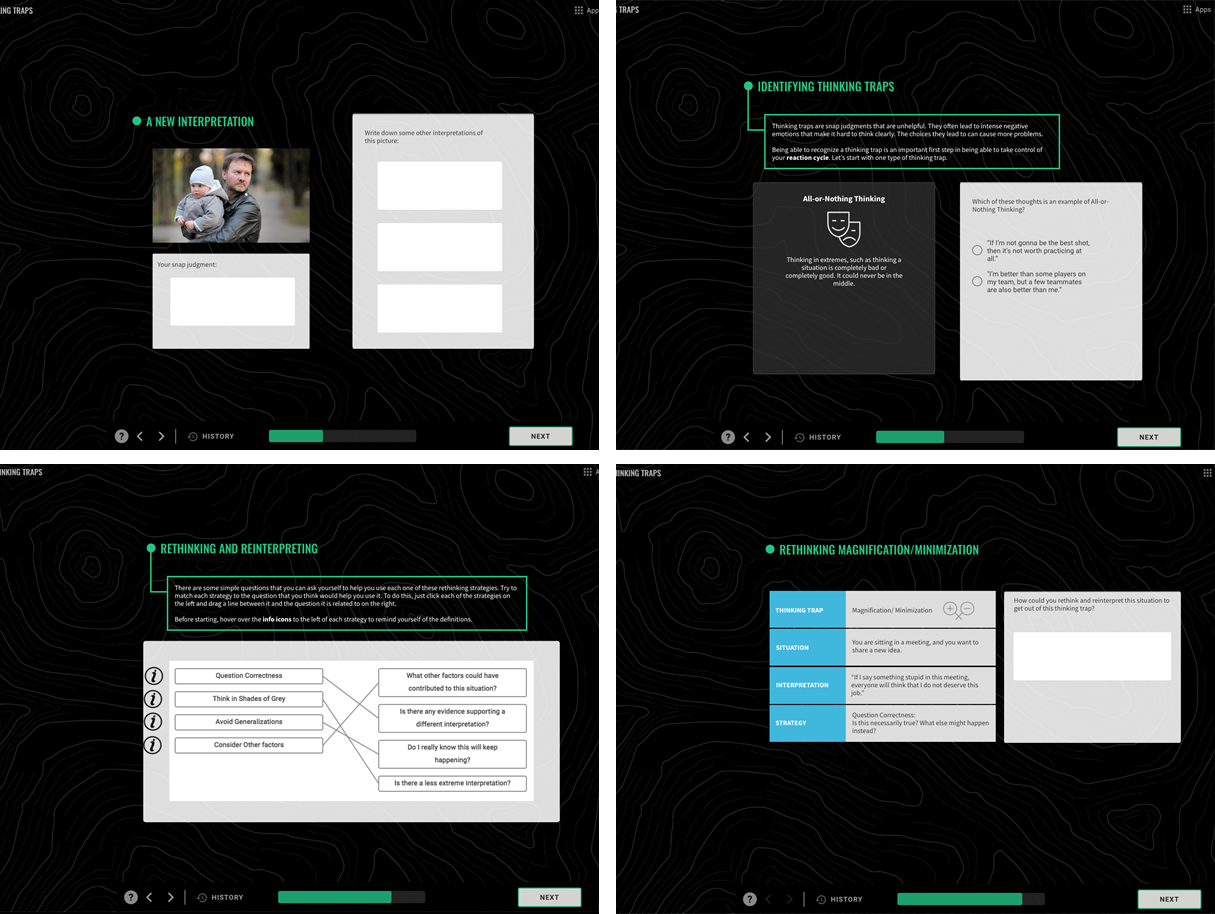


*Figure 7*. Activity encouraging participants to reinterpret their initial judgment of a situation (top left); example of a cognitive distortion (top right); activity demonstrating strategies to challenge cognitive distortions (bottom left); application exercise for rethinking and reinterpreting a situation (bottom right).

Images reproduced with permission from Maria Sbytova/shutterstock.com

**Module Aims**: This module teaches and allows the participant to practice an effective emotion regulation technique called cognitive reappraisal.

**Targeted Knowledge, Skills, and Abilities**: Identifying thinking traps (cognitive distortions), improving flexibility in thinking, changing unhelpful thoughts by rethinking and reinterpreting them.

**Description of Activities**: Opening emotion tracker exercise, overview of thinking traps and how they impact the reaction cycle, exercise for reinterpreting situations, exercise matching thoughts to the correct cognitive distortion, exercise matching the cognitive distortions to effective ways of correcting the distortion, application activity for rethinking and reinterpreting a situation containing distortions.

**Average Completion Time**: 31 minutes

**Schedule:** Day 3 (Compressed); Day 9 (Distributed)

**Module 7: Recognizing Emotions**


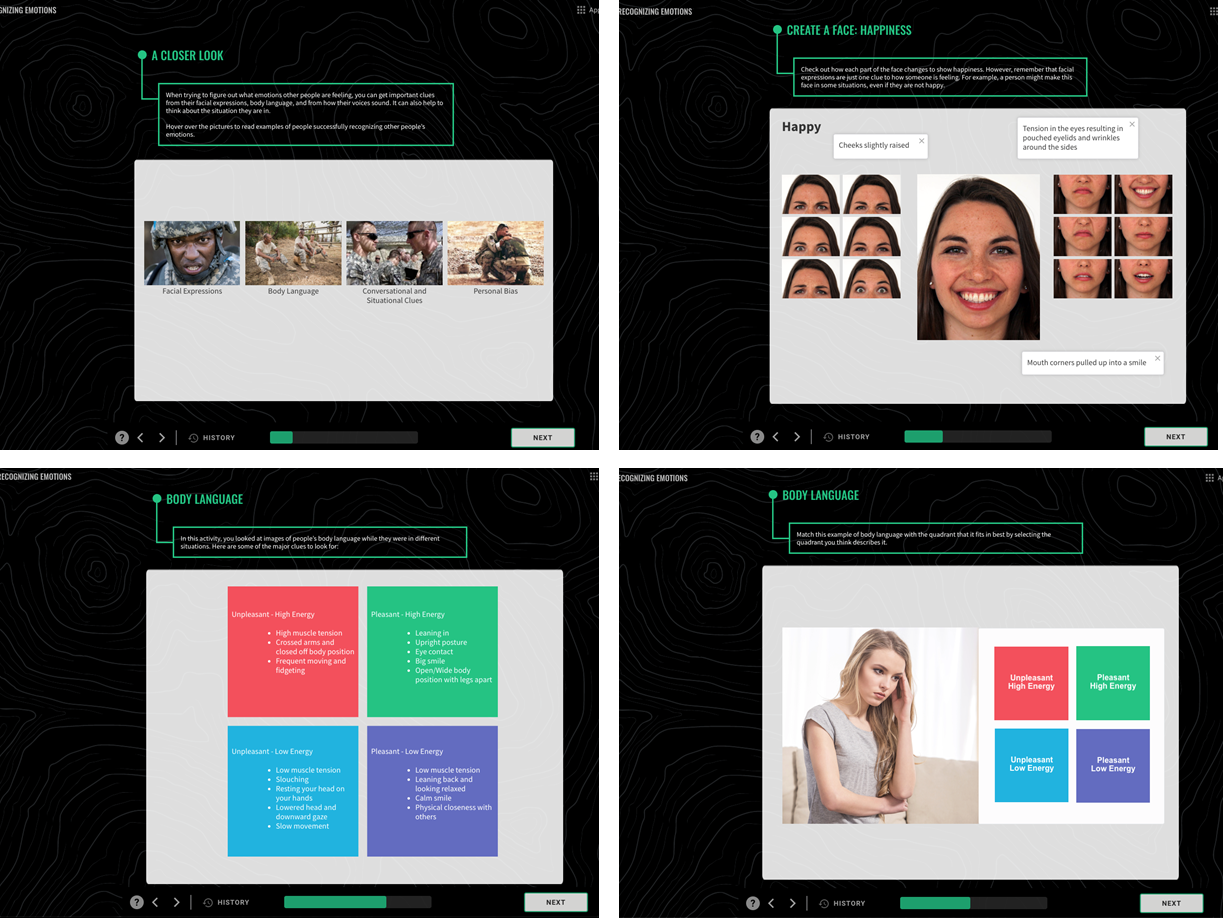


*Figure 8*. Description of different channels for recognizing others’ emotions (top left); illustration of facial features that make a happy expression (top right); description of body cues that convey different emotions (bottom left); application of body cue knowledge (bottom right).

Images reproduced with permission from VGstockstudio/shutterstock.com.

**Module Aims**: This model teaches participants how to recognize emotions and how to differentiate emotional signals in different contexts. This includes practice recognizing emotional signals in both faces and body language, based on specific cues.

**Targeted Knowledge, Skills, and Abilities**: Paying attention to other people’s behaviors, improving ability to perceive and understand what other people are thinking and feeling, noticing when one’s own thoughts and feelings are influencing one’s perception of others.

**Description of Activities:** Opening emotion tracker exercise, demonstration of facial expression for basic emotions, demonstration of body language associated with different types of emotions, interactive scenario demonstrating how one’s emotional state can color perceptions of others’ emotions.

**Average Completion Time**: 26 Minutes

**Schedule:** Day 4 (Compressed); Day 10 (Distributed)

**Module 8: Emotions and Behaviors**


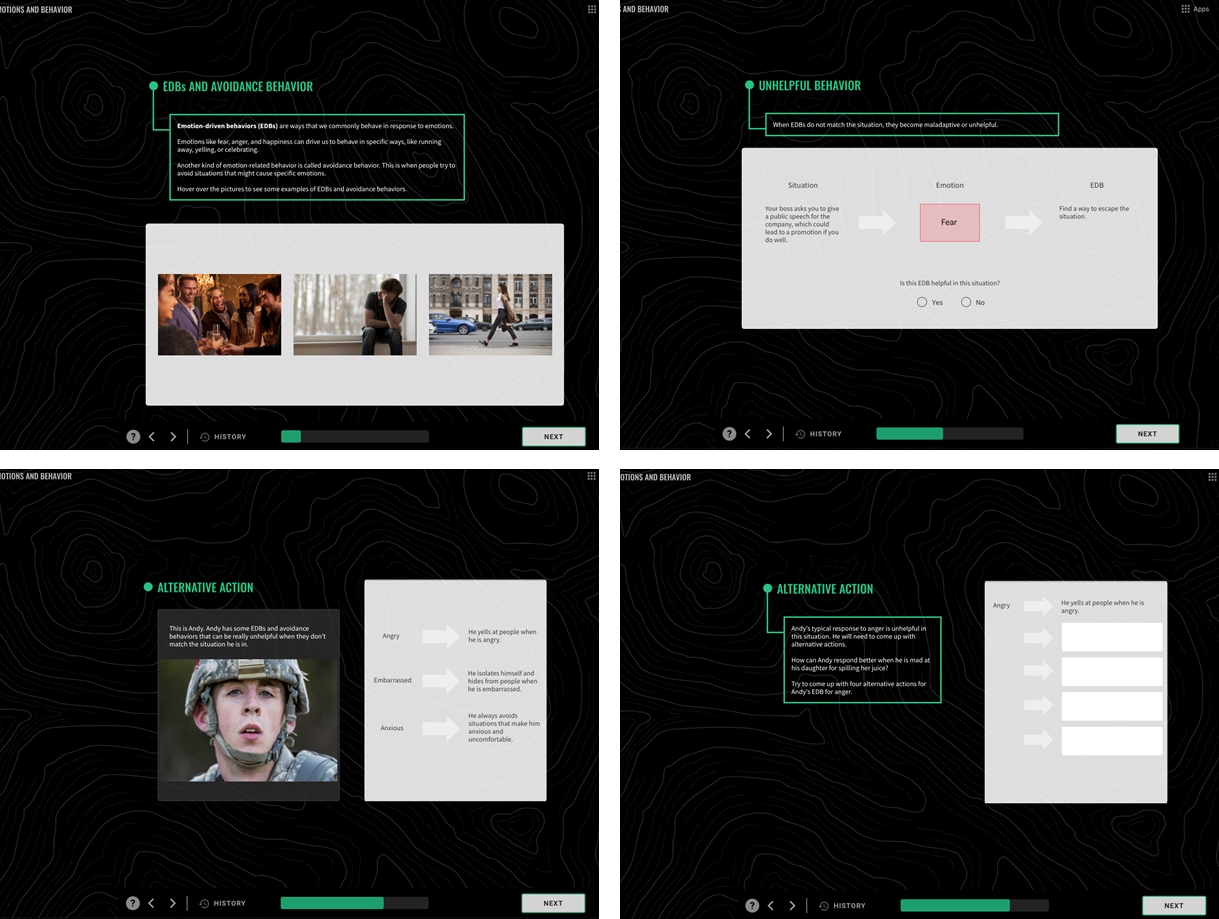


*Figure 9*. Description and examples of emotion-driven behavior (top left); Example of a maladaptive emotion-driven behavior (top right); example of a person’s emotion-driven and avoidance behaviors (bottom left); application activity encouraging participants to generate alternatives to a maladaptive emotion-driven behavior (bottom right).

Images reproduced with permission from Andrey Arkusha/shutterstock.com; Ground Pictures/shutterstock.com; Monkey Business Images/shutterstock.com.

**Module Aims**: This module teaches participants how to recognize, and if necessary, change the behaviors that are automatically provoked by strong emotions.

**Targeted Knowledge, Skills, and Abilities:** Recognizing the connection between emotions and behaviors, learning when emotion driven and avoidance behaviors can be helpful or unhelpful, learning how to take alternative actions to maladaptive behaviors provoked by emotion.

**Description of Activities:** Opening emotion tracker activity, overview of emotion-driven and avoidance behaviors, activity identifying whether a behavior is adaptive or maladaptive given the context, activity generating more adaptive behaviors for responding to strong emotions.

**Average Completion Time**: 26 minutes

**Schedule:** Day 4 (Compressed); Day 11 (Distributed)

**Module 9: Responding to Others**


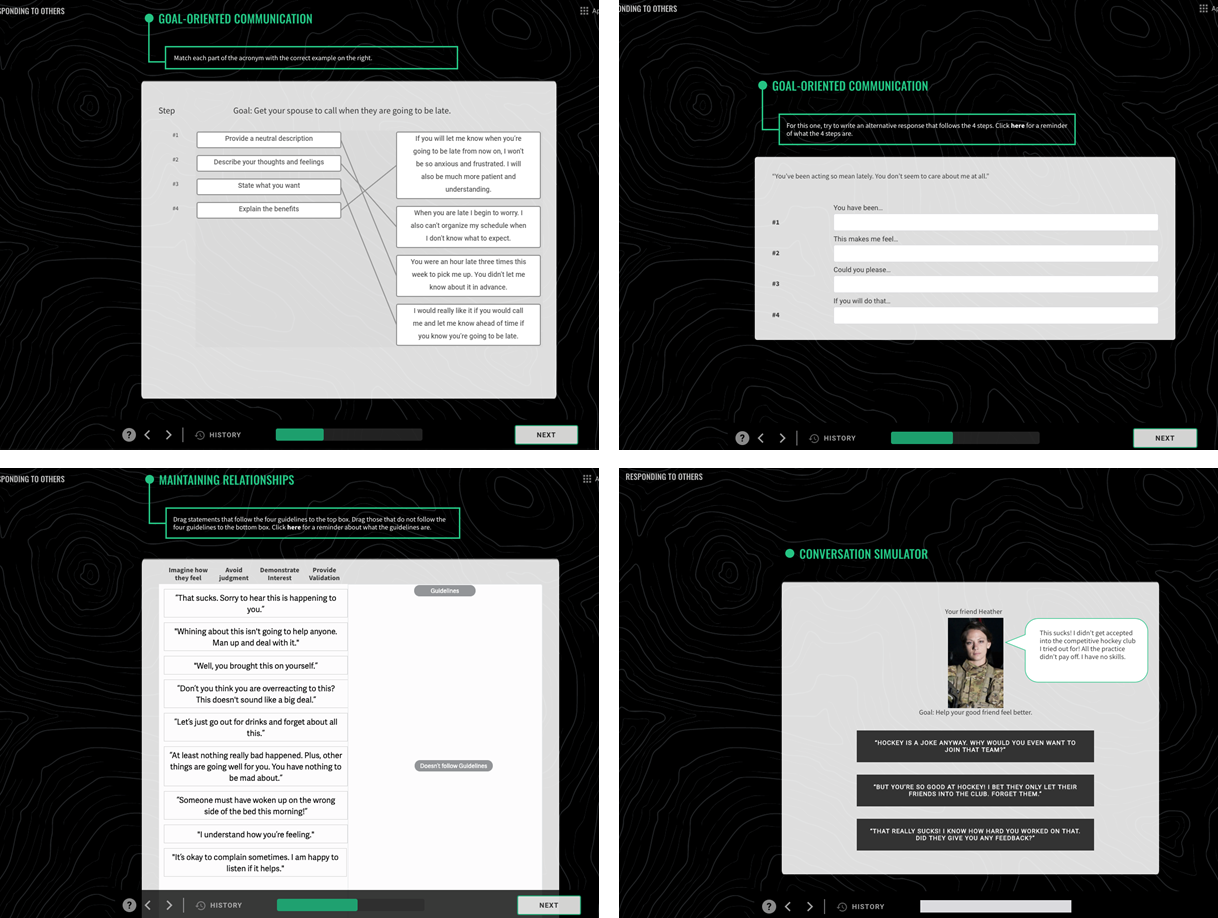


*Figure 10*. Activity applying goal-oriented communication practices (top left); activity generating responses to a scenario (top right); activity identifying helpful and nonhelpful ways of responding to a person (bottom left); activity applying communication skills to specific situations (bottom right).

All images were reproduced under the Department of Defense public domain.

**Module Aims**: This module teaches participants how to recognize the way they often respond to others in different social situations, including those within personal and professional relationships.

**Targeted Knowledge, Skills, and Abilities:** Learning guidelines for effective communication, effectively interacting with others as a way to achieve one’s own goals, empathizing with others to help maintain and strengthen relationships.

**Description of Activities:** Opening emotion tracker activity, overview of the benefits of effectively responding to others, description of guidelines for effective goal-oriented communication and practice applying guidelines, description of guidelines for effective relationship-maintaining communication and practice applying guidelines, conversation simulator activity for applying skills learned in the module.

**Average Completion Time**: 47 minutes

**Schedule:** Day 5 (Compressed); Day 13 (Distributed)

**Module 10 (Part 1): Relaxation and Mindfulness**


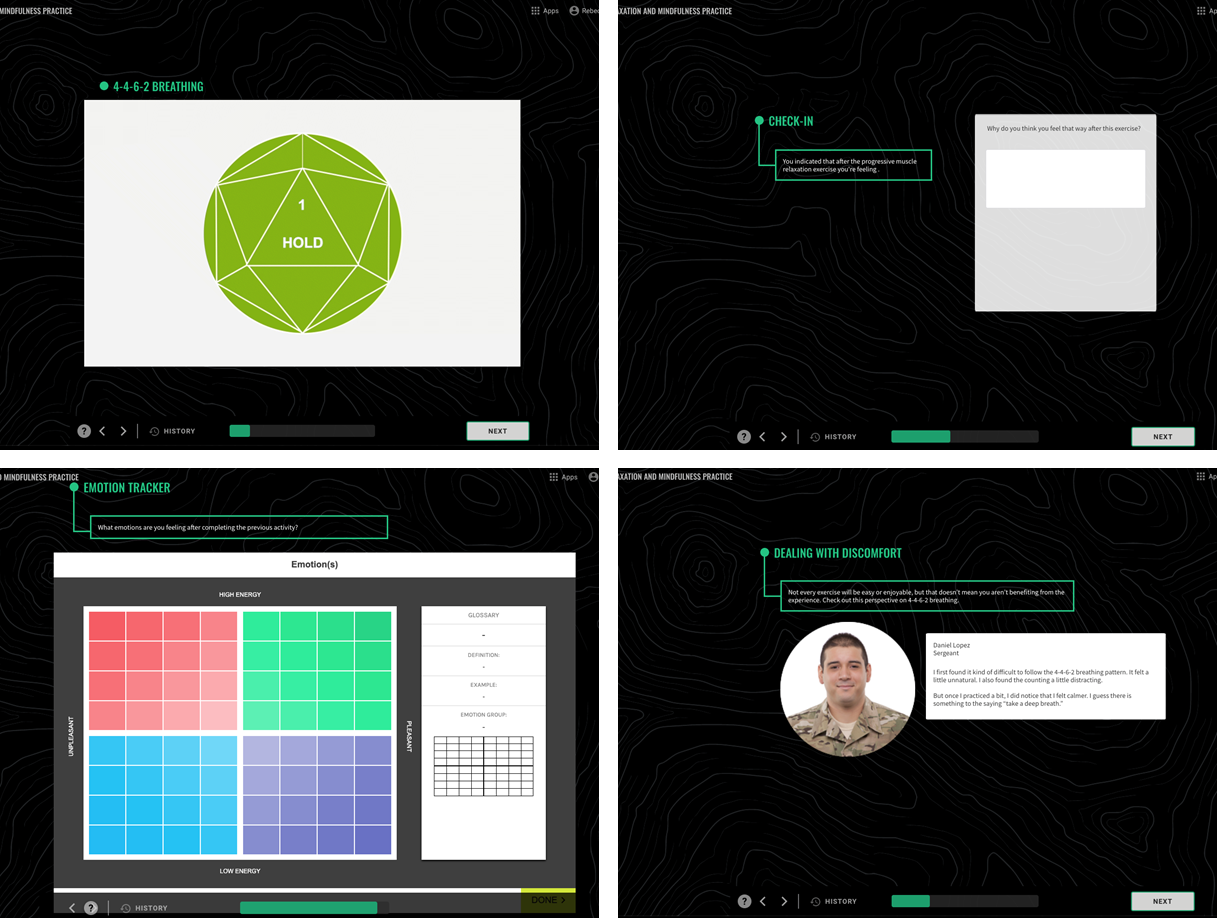


*Figure 11*. Display for a mindfulness-based breathing exercise (top left); free response reflection on how participant felt during the mindfulness exercise (top right); emotion tracker reflection on what emotions the participant felt during the mindfulness exercise (bottom left); discussion of managing feelings of discomfort during exercise (bottom right).

Images reproduced with permission from Straight 8 Photography/shutterstock.com.

**Module Aims**: This module provides extended practice and training exercises and feedback to improve mindfulness and relaxation skills taught earlier in the program.

**Targeted Knowledge, Skills, and Abilities**: Mindfulness and relaxation, paying attention to emotional reactions, addressing common negative reactions (e.g., discomfort) to mindfulness.

**Description of Activities**: 4-4-6-2 breathing pattern exercise, progressive muscle relaxation exercise, guided body scan exercise, mindful attention to emotion exercise, mindful listening exercise.

**Average Completion Time**: 60 minutes

**Schedule:** Day 6 (Compressed); Day 15 (Distributed)

**Module 10 (Part 2): Challenge 1**


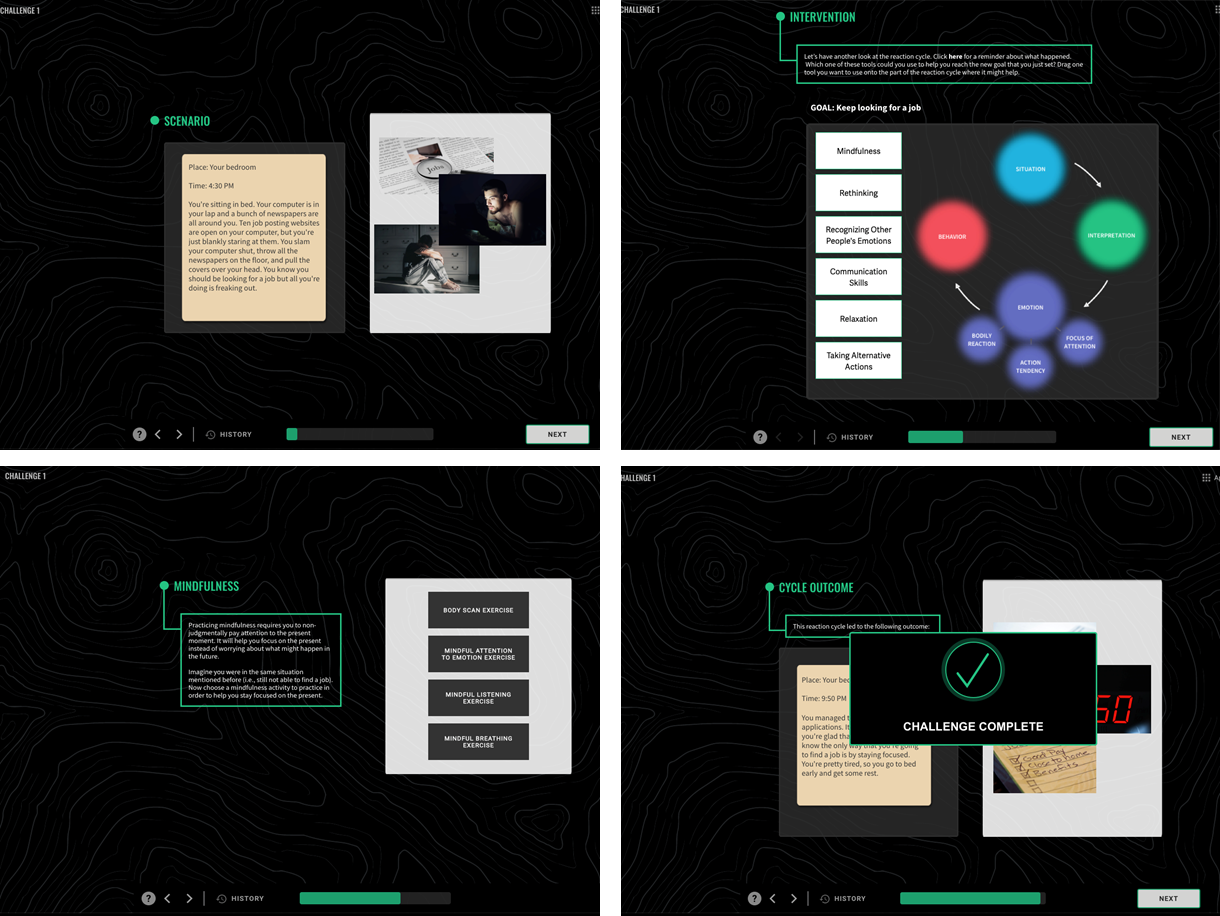


*Figure 12*. Scenario for the challenge (top left); applying skills learning in training program to help resolve the scenario (top right); applying mindfulness to help with the scenario (bottom left); completion after successfully using mindfulness and other skills to achieve the challenge goal (bottom right).

Images reproduced with permission from rawf8shutterstock.com; konzeptm/shutterstock.com; Melinda Fawver/shutterstock.com; Estrada Anton/shutterstock.com; bluedog studio/shutterstock.com.

**Module Aims**: This module provides an opportunity to apply skills learned throughout the program in order to successfully resolve a scenario.

**Targeted Knowledge, Skills, and Abilities:** Ability to apply the mindfulness skills and other emotion-related knowledge learned in the program to successfully resolve a situation.

**Description of Activities:** Interactive scenario in which one must apply mindfulness skills and other skills from the program to achieve a desired goal.

**Average Completion Time**: 22 minutes

**Schedule:** Day 6 (Compressed); Day 15 (Distributed)

**Module 11 (Part 1): Flexible Thinking**


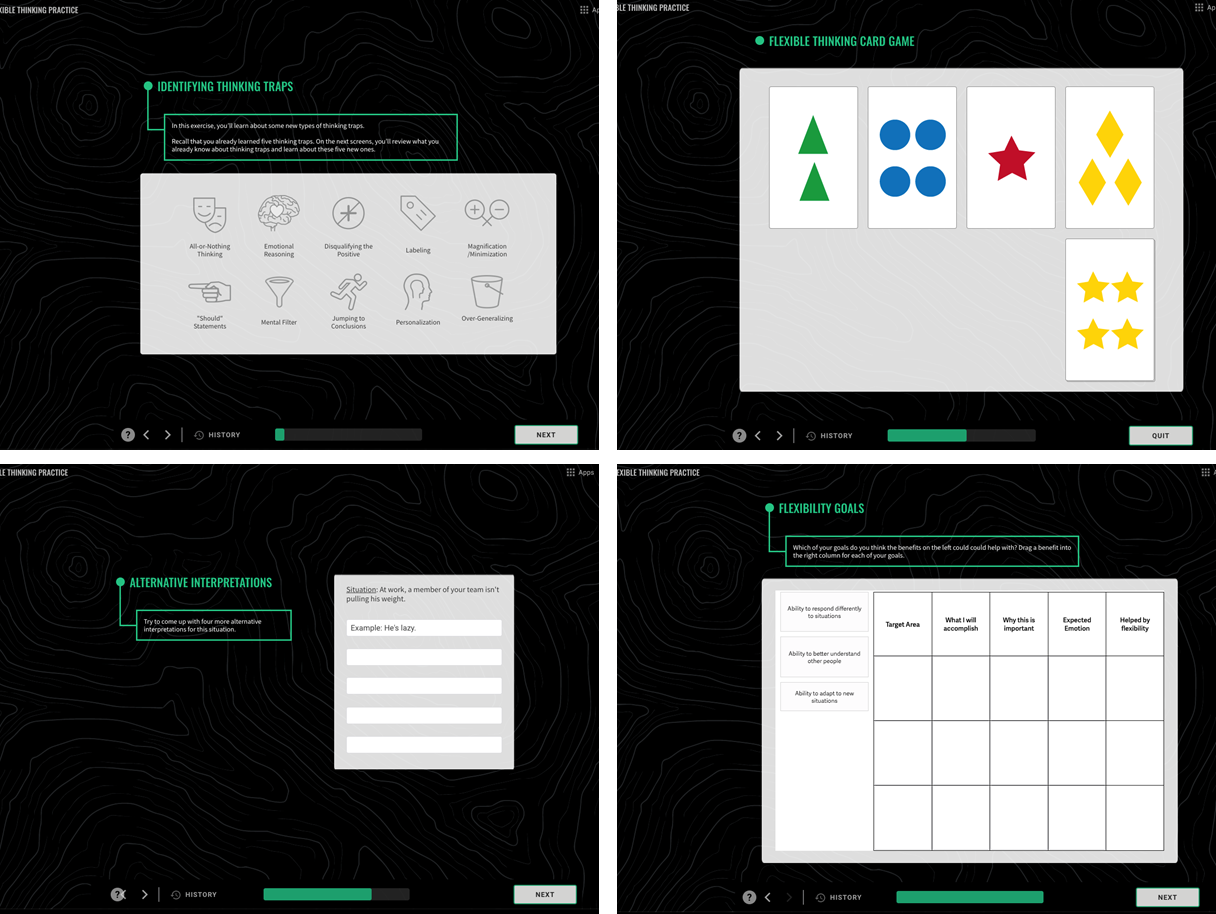


*Figure 13*. Description of cognitive distortions (top left); a flexible thinking game (top right); application activity generating alternative interpretation for a behavior (bottom left); how flexible thinking can help achieve goals (bottom right).

All images and content were produced by the University of Arizona.

**Module Aims**: This module provides extended practice and training exercises and feedback to improve cognitive flexibility and reappraisal skills taught earlier in the program.

**Targeted Knowledge, Skills, and Abilities**: Identifying thinking traps (cognitive distortions), practicing flexible thinking, reinterpreting situations and behaviors.

**Description of Activities:** Activity identifying thinking traps and connecting example thoughts to the correct trap, flexible thinking card game, flexible thinking computer game, activity generating alternative interpretations to situations and behaviors.

**Average Completion Time**: 36 minutes

**Schedule:** Day 6 (Compressed); Day 16 (Distributed)

**Module 11 (Part 2): Challenge 2**


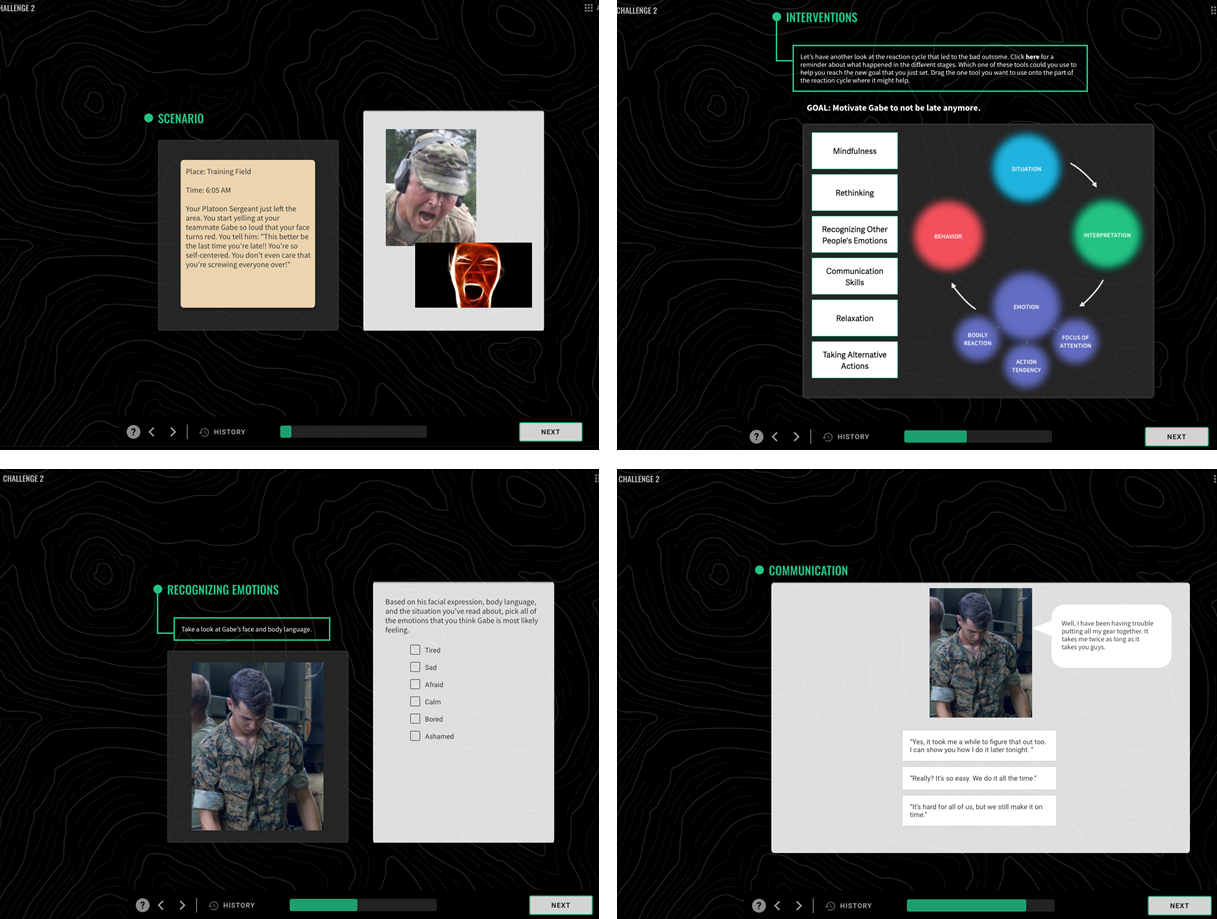


*Figure 14*. Scenario for the challenge (top left); applying skills from the program to resolve the scenario (top right); applying emotion recognition skills to help effectively resolve the scenario (bottom left); applying communication skills to help effectively resolve the scenario (bottom right).

All images accessed and reproduced under the Department of Defense public domain.

**Module Aims:** This module provides an opportunity to apply skills learned throughout the program in order to successfully resolve a scenario.

**Targeted Knowledge, Skills, and Abilities:** Ability to apply the flexible thinking skills and other emotion-related knowledge learned in the program to successfully resolve a situation.

**Description of Activities:** Interactive scenario in which one must apply flexible thinking skills and other skills from the program to achieve a desired goal.

**Average Completion Time**: 12 minutes

**Schedule:** Day 6 (Compressed); Day 16 (Distributed)

**Module 12 (Part 1): Social Skills**


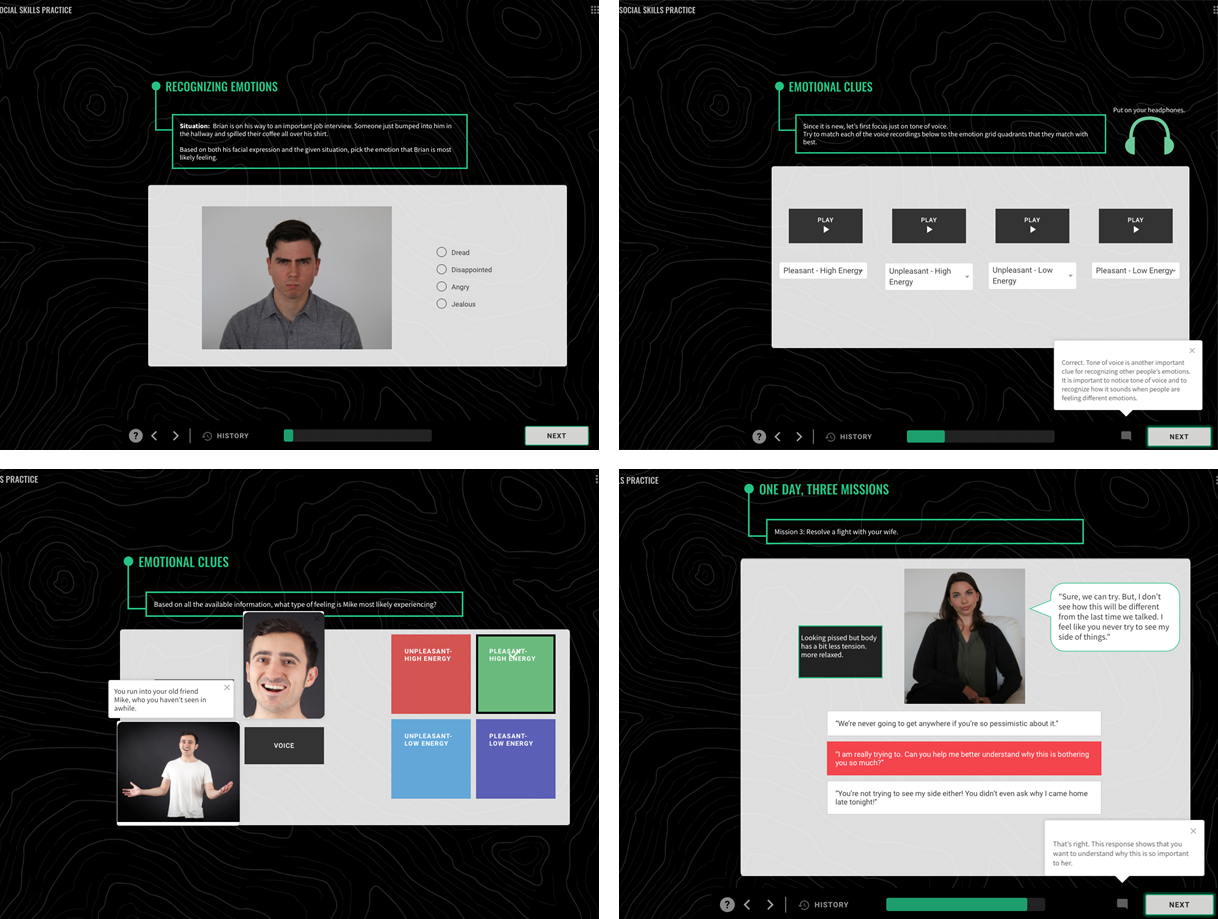


*Figure 15*. Practice recognizing emotion based on situation and expression (top left); practice recognizing emotions based on verbal cues (top right); practice recognizing emotion based on situation, expression, body cues, and voice (bottom left); applying emotion recognition and communication skills to resolve a scenario (bottom right).

Images reproduced with permission from Pixabay.

**Module Aims**: This module provides extended practice and training exercises and feedback to improve the emotion recognition abilities and social skills taught earlier in the program.

**Targeted Knowledge, Skills, and Abilities:** Recognizing emotions in facial expressions, recognizing emotions using vocal cues, recognizing emotions using multi-modal cues, recognizing emotions using conversational cues, effectively communicating with others.

**Description of Activities:** Practice identifying emotions based on expression and context, practice identifying emotions based on facial, verbal, body, and context clues, interactive scenario using emotion recognition skills to have a successful conversation, role play activity to practice empathetic responding, conversation simulator to practice effective communication.

**Average Completion Time**: 38 minutes

**Schedule:** Day 7 (Compressed); Day 17 (Distributed)

**Module 12 (Part 2): Challenge 3**


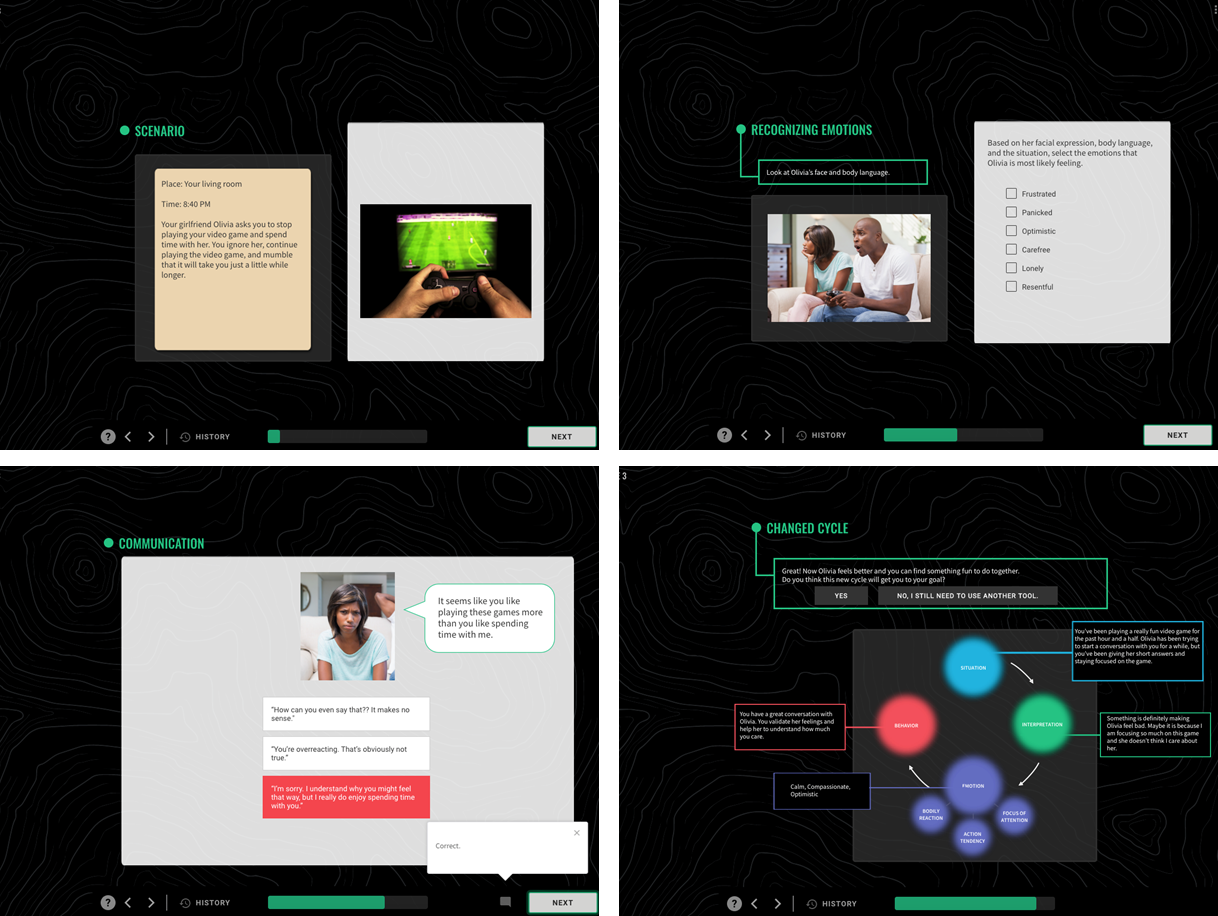


*Figure 16*. Scenario for the challenge (top left); applying emotion recognition skills to help effectively resolve the scenario (top right); applying communication skills to help effectively resolve the scenario (bottom left); demonstration of how the applied skills helped improve the reaction cycle.

Images reproduced with permission from Georgiy M/shutterstock.com; wavebreakmedia/shutterstock.com.

**Module Aims:** This module provides an opportunity to apply skills learned throughout the program in order to successfully resolve a scenario.

**Targeted Knowledge, Skills, and Abilities:** Ability to apply the social skills and other emotion-related knowledge learned in the program to successfully resolve a situation.

**Description of Activities:** Interactive scenario in which one must apply social skills and other skills from the program to achieve a desired goal.

**Average Completion Time**: 13 minutes

**Schedule:** Day 7 (Compressed); Day 17 (Distributed)

**Module 13: Self-Investigation**


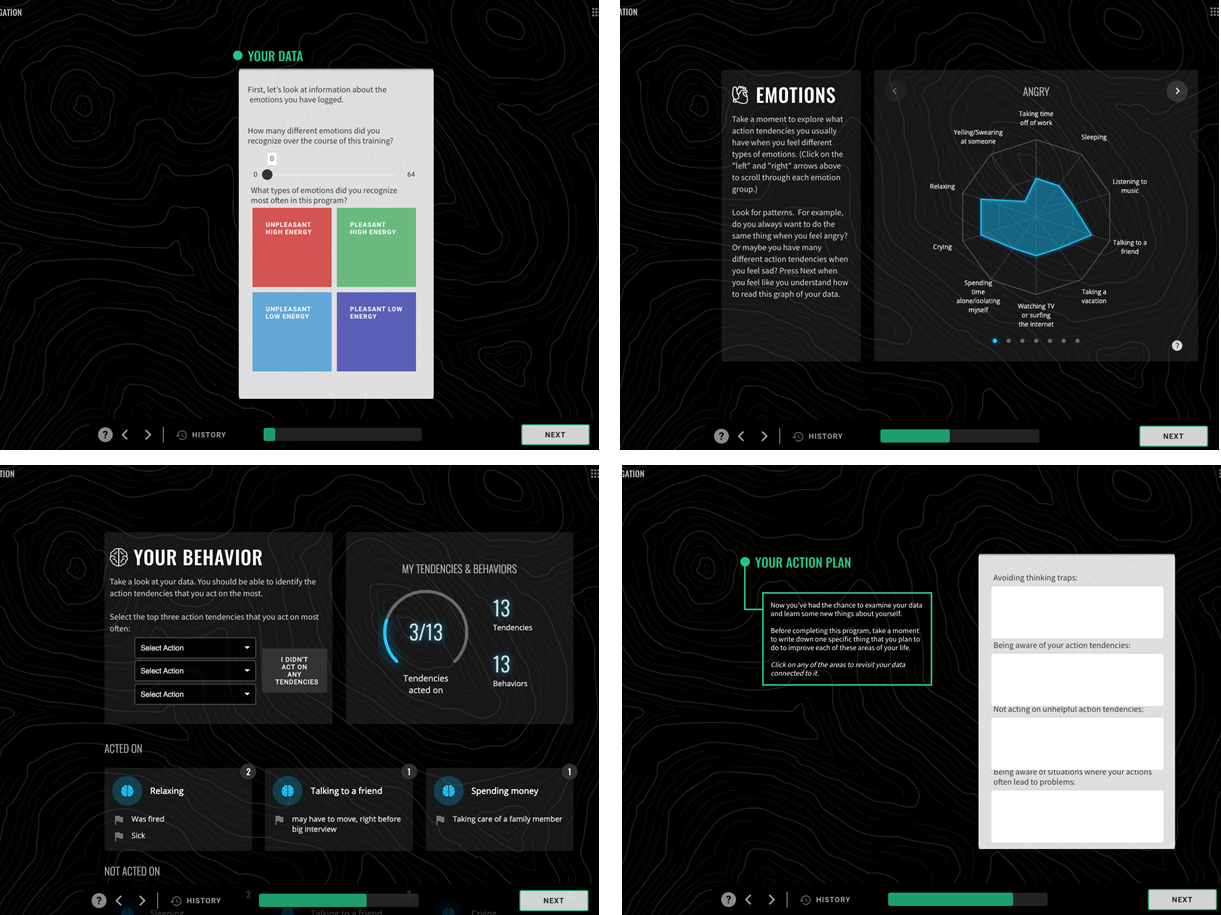


*Figure 17*. Example of the different types of emotions a person experienced during the program (top left); example of a summary of a participant’s action tendencies when they feel angry (top right); example of action tendency summary (bottom left); goals for incorporating lessons learned in the program (bottom right).

All images and content produced by the University of Arizona.

**Module Aims**: Presents the participant with all of their data gathered throughout the program, including their different recorded emotions, situations, and responses, and how much their skills increased during the program.

**Targeted Knowledge, Skills, and Abilities:** Identifying patterns in one’s responses throughout training, awareness of problem areas and how to address them, and motivation to continue practice and keep improving the skills learned in the program.

**Description of Activities:** Presentation of the person’s data collected throughout the program, self-reflection activities.

**Average Completion Time**: 32 minutes

**Schedule:** Day 7 (Compressed); Day 18 (Distributed)
